# Supplementary material for: Machine learning health-related applications in low-income and middle-income countries: a scoping review protocol
Source: BMJ Open. 2020 May 10;10(5):e035983. doi: 10.1136/bmjopen-2019-035983 (PMC7223147; doi:10.1136/bmjopen-2019-035983)
Supplement: Author's manuscript [file bmjopen-2019-035983.draft_revisions.pdf]

BMJ Open is committed to open peer review. As part of this commitment we make the peer review history of every article we publish publicly available.

When an article is published we post the peer reviewers' comments and the authors' responses online. We also post the versions of the paper that were used during peer review. These are the versions that the peer review comments apply to.

The versions of the paper that follow are the versions that were submitted during the peer review process. They are not the versions of record or the final published versions. They should not be cited or distributed as the published version of this manuscript.

BMJ Open is an open access journal and the full, final, typeset and author-corrected version of record of the manuscript is available on our site with no access controls, subscription charges or pay-per-view fees (http://bmjopen.bmj.com).

If you have any questions on BMJ Open's open peer review process please email info.bmjopen@bmj.com

BMJ Open

Machine learning health-related applications in low- and middle-income countries: A scoping review protocol

Journal:	*BMJ Open*
Manuscript ID	bmjopen-2019-035983
Article Type:	Protocol
Date Submitted by the Author:	25-Nov-2019

Complete List of Authors:	Carrillo-Larco, Rodrigo; Universidad Peruana Cayetano Heredia, CRONICAS Center of Excellence; CRONICAS Center of Excellence in Chronic Diseases<br>Tudor Car, Lorainne; Lee Kong Chian School of Medicine<br>Pearson-Stuttard, Jonathan; Imperial College London, PCPH<br>Panch, Trishan<br>Miranda, J. Jaime; Universidad Peruana Cayetano Heredia, CRONICAS Centre of Excellence in Chronic Diseases<br>Atun, Rifat; Harvard University, Harvard School of Public Health

Keywords:	EPIDEMIOLOGY, BIOTECHNOLOGY & BIOINFORMATICS, Health informatics < BIOTECHNOLOGY & BIOINFORMATICS, World Wide Web technology < BIOTECHNOLOGY & BIOINFORMATICS

SCHOLARONE™
Manuscripts

I, the Submitting Author has the right to grant and does grant on behalf of all authors of the Work (as defined in the below author licence), an exclusive licence and/or a non-exclusive licence for contributions from authors who are: i) UK Crown employees; ii) where BMJ has agreed a CC-BY licence shall apply, and/or iii) in accordance with the terms applicable for US Federal Government officers or employees acting as part of their official duties; on a worldwide, perpetual, irrevocable, royalty-free basis to BMJ Publishing Group Ltd ("BMJ") its licensees and where the relevant Journal is co-owned by BMJ to the co-owners of the Journal, to publish the Work in this journal and any other BMJ products and to exploit all rights, as set out in our licence.

The Submitting Author accepts and understands that any supply made under these terms is made by BMJ to the Submitting Author unless you are acting as an employee on behalf of your employer or a postgraduate student of an affiliated institution which is paying any applicable article publishing charge ("APC") for Open Access articles. Where the Submitting Author wishes to make the Work available on an Open Access basis (and intends to pay the relevant APC), the terms of reuse of such Open Access shall be governed by a Creative Commons licence – details of these licences and which Creative Commons licence will apply to this Work are set out in our licence referred to above.

Other than as permitted in any relevant BMJ Author's Self Archiving Policies, I confirm this Work has not been accepted for publication elsewhere, is not being considered for publication elsewhere and does not duplicate material already published. I confirm all authors consent to publication of this Work and authorise the granting of this licence.

For peer review only

Machine learning health-related applications in low- and middle-income countries: A scoping review protocol

Rodrigo M Carrillo-Larco (r.carrillo-larco@imperial.ac.uk) [1,2]

Lorainne Tudor Car (lorainne.tudor.car@ntu.edu.sg) [3,4]

Jonathan Pearson-Stuttard (j.pearson-stuttard@imperial.ac.uk) [5]

Trishan Panch (trishan@wellframe.com) [6]

J Jaime Miranda (jaime.miranda@upch.pe) [2,7]

Rifat Atun (ratun@hsph.harvard.edu) [8]

1. Department of Epidemiology and Biostatistics, School of Public Health, Imperial College London, London, UK.
2. CRONICAS Centre of Excellence in Chronic Diseases, Universidad Peruana Cayetano Heredia, Lima, Peru.
3. Family Medicine and Primary Care, Lee Kong Chian School of Medicine, Nanyang Technological University, Singapore.
4. Department of Primary Care and Public Health, School of Public Health, Imperial College London, London, UK.
5. Department of Epidemiology and Biostatistics and MRC-PHE Centre for Environment and Health, School of Public Health, Imperial College London, London, UK.
6. Wellframe Inc., Boston, Massachusetts, USA.
7. Facultad de Medicina "Alberto Hurtado", Universidad Peruana Cayetano Heredia, Lima, Peru.
8. Harvard T.H Chan, School of Public Health, and Harvard Medical School, Harvard University, Cambridge, Massachusetts, USA.

For peer review only - http://bmjopen.bmj.com/site/about/guidelines.xhtml

Corresponding author:

Rodrigo M. Carrillo-Larco, MD

Department of Epidemiology and Biostatistics

School of Public Health

Imperial College London, London, UK

r.carrillo-larco@imperial.ac.uk

Conflict of interest: none to declare.

Funding: RMC-L has been supported by a Strategic Award, Wellcome Trust-Imperial College Centre for Global Health Research (100693/Z/12/Z), and Imperial College London Wellcome Trust Institutional Strategic Support Fund [Global Health Clinical Research Training Fellowship] (294834/Z/16/Z ISSF ICL). RMC-L is supported by a Wellcome Trust International Training Fellowship (214185/Z/18/Z). The funders had no role in this work and decision to submit for publication.

Key words: Artificial intelligence; Algorithms; Developing countries

Word count: 1,540

For peer review only - http://bmjopen.bmj.com/site/about/guidelines.xhtml

ABSTRACT

Introduction: Machine learning (ML) has been used in bio-medical research, and recently in clinical and public health research. However, much of the available evidence comes from high-income countries where different health profiles challenges the application of this research to low- and middle-income countries (LMICs). It is largely unknown what ML applications are available for LMICs that can support and advance clinical medicine and public health. We aim to address this gap by conducting a scoping review of health-related ML applications in LMICs.

Methods and analysis: This scoping review will follow the methodology proposed by Levac *et al*. The search strategy is informed by recent systematic reviews of ML health-related applications. We will search Embase, Medline and Global Health (through OVID), Cochrane and Google Scholar. Titles and abstracts will be screened by two reviewers independently; selected reports will be studied by two reviewers independently. Reports will be included if they are primary research where data have been analysed, ML techniques have been used on data from LMICs, and they aimed to improve health-related outcomes. We will synthetize the information following evidence mapping recommendations.

Ethics and dissemination: The review will provide a comprehensive list of health-related ML applications in LMICs. The results will be disseminated through scientific publications. We also plan to launch a website where ML models can be hosted so that researchers, policymakers and the general public can readily access them.

For peer review only

For peer review only - http://bmjopen.bmj.com/site/about/guidelines.xhtml

STRENGTHS AND LIMITATIONS OF THIS STUDY

- This protocol outlines a comprehensive scoping review to map machine learning health-related applications for low- and middle-income countries, an enterprise not conducted before.
- The search strategy is informed by a solid framework, the search will be conducted in several information sources, and the screening/review will be performed by two reviewers independently.
- As part of the dissemination plan, we aim to launch a website where the results and original machine learning models can be readily accessed, fostering the using and further research of machine learning applications in low- and middle-income countries.
- We will not search abstracts or conference proceedings specialized in machine learning, focusing on information sources where health-related outcomes are most likely to be found.

For peer review only

INTRODUCTION

Machine learning refers to the process through which computers, models or algorithms, learn and improve from data and processes, rather than from specific programmed instructions.[1,2] Machine learning can be used in tasks such as classification (e.g., whether a tumour is benign or malign based on patterns or characteristics), clustering (e.g., group patients with similar profiles for targeted prevention or treatment interventions) or prediction (e.g., forecast propensity to risk or probability of outcome of a disease following interventions).[2]

Machine learning is widely used in bio-medical sciences, and more recently in clinical and public health research.[3,4] Systematic reviews on health-related applications of machine learning have explored questions such as the accuracy of machine learning for diagnosis or outcome prediction,[5-7] but most of the research studies included in these reviews have come from high-income countries and the findings may not apply to low-income and middle-income countries (LMICs) because the variability in access to healthcare and difference in the disease burden. It is largely unknown what machine learning applications are available for LMICs that can support and advance clinical medicine and public health.

Our aim was to address this gap in the evidence by conducting a scoping review of health-related applications of machine learning in LMICs to synthesise published evidence and to garner lessons to inform further research and policy development. The review will provide the first comprehensive list of health-related machine learning applications in LMICs.

METHODS

Overview

This is a scoping review of the published scientific literature. This protocol adheres to the PRISMA-P guidelines (supplementary material S1),[8] and the methodology follows the procedures suggested by Levac et al.[9] The final publication of this work will adhere to the PRISMA-ScR recommendations.[10]

For peer review only - http://bmjopen.bmj.com/site/about/guidelines.xhtml

For peer review only

Understanding that a key feature of scoping reviews is a broad research question, we aim to answer: *what have been the health-related applications of machine learning techniques in LMICs?* This review will summarize scientific evidence on research that have used data from LMICs for machine learning applications in clinical medicine (e.g., risk prediction for clinical decisions) and public health (e.g., vector control in an endemic area) to provide solutions to health problems in LMICs.

Definitions

For this work we will follow these definitions:

- LMICs: classification by the World Bank country income grouping (see search strategy in Table 1 for a list of countries).[11]
- Machine learning: analytical techniques through which computers learn directly from data, examples and experiences, rather than from a pre-programmed rule.[1,2]
- Machine learning techniques: analytical methods within the machine learning remit, i.e. where machines have learnt from data or processes through machine learning techniques or algorithms. These include, but are not limited to:[2,12] support vector machine, support vector regression, decision trees, random forest, neural network, Bayesian network, artificial neural network, computer vision systems, computer-assisted image processing and natural language processing.

Eligibility criteria

We will include scientific evidence that meets the following inclusion criteria (Figure 1):

Primary research studies.

We will include experimental and observational studies that have used and report quantitative data. We will also screen relevant systematic reviews or narrative reviews for eligible primary studies and include them in our review if relevant. We will not include qualitative studies, opinions, conference

For peer review only - http://bmjopen.bmj.com/site/about/guidelines.xhtml

abstracts, letters, editorials or any other scientific work in which data have not been actively analysed within a machine learning framework.

Machine learning research should have used data from LMICs, i.e. machine learning research that used solely data from LMICs.

We will focus on machine learning research based on data from LMICs applied to LMICs. We will not include machine learning research that used data from high-income countries even though it could have been applied to a LMIC, and neither studies using LMICs data applied to high-income countries. This scoping review focuses on the applications of machine learning techniques in LMICs developed using LMICs data as prediction models and other algorithms work better, i.e. have better accuracy, in the population – data – for which they have been developed. Conversely, when these models are applied to other populations, settings or data, they need some modification (e.g., re-calibration). We will also exclude studies that have used LMIC data in a consortium or data pooling group if the model or results cannot be separated for the LMIC alone; in other words, we will exclude a report if this used LMIC data in aggregate with data from high-income countries, but the application cannot be separated for the LMIC alone.

Examples of studies of interest include: i) development of a 'deep learning-based visual evaluation algorithm' to early identify cervical cancer signs based on data from women in Costa Rica.[13] ii) Classification of free-text (random forest) in emergency department records from nine hospitals in Nicaragua.[14] iii) Automatic classification (neural networks) of paediatric pneumonia based on ultrasound records from children in Peru.[15] During the selection stages.

The outcome of the study/analysis was to improve a health-related outcome.

The *primary outcome* of the selected studies should have sought improvements in the following health-related endpoints along the care cascade: diagnosis, treatment, control, survival, complications and mortality. These outcomes have been selected because of their relevance in healthcare, clinical

For peer review only - http://bmjopen.bmj.com/site/about/guidelines.xhtml

medicine and public health. As *secondary outcome* we will also include studies that have reported endpoints related to cost, efficiency and productivity in the healthcare process.

Information sources

We will conduct the search in three databases through Ovid: Embase, Medline and Global Health; in addition, we will search Cochrane and Google Scholar (first 10 pages). These sources will be used without language or time/year restrictions.

Search strategy

Based on recent systematic reviews of machine learning applications on health-related endpoints: outcome prediction in gastrointestinal bleeding,[7] assessment of physicians knowledge,[16] applications on genomic data to predict outcomes in cancer patients,[17] and available machine learning algorithms to improve genomic data analysis,[18] we developed our search strategy (Table 1).. We will screen the references of the included reports for any other relevant studies.

Study records

Results will be downloaded into EndNote where duplicates will be omitted, and a second cleaning of duplicated results will be conducted using the online tool Rayyan.[19] Titles and abstracts will be reviewed by two independent researchers following the above detailed selection criteria (Figure 1); discrepancies will be solved by consensus or by a third party. Selected titles will be sought in full-text, which will be assessed by two independent reviewers following the same selection criteria (Figure 1). Again, in case of conflicts, these will be solved by consensus or by a third party.

Data collection

The reviewers will decide on a list of items that will be extracted to successfully answer the research question. These items will be implemented in an Excel spreadsheet before data extraction and will not be modified afterwards. Data from the selected reports will be extracted by two researchers

For peer review only - http://bmjopen.bmj.com/site/about/guidelines.xhtml

independently; if there were discrepancies, these will be solved by consensus among them or by a third party.

Risk of bias in individual studies

Because this is a scoping review aiming to summarise available evidence to identify research gaps and potential uses of machine learning to improve health-related outcomes in LMICs, no risk of bias of individual studies is planned.

Data synthesis

We anticipate a large heterogeneity of selected reports, both in terms of methodology and outcomes, as well as target population and data sources. Therefore, no meta-analysis is planned and only a qualitative summary will be conducted. Following current recommendations for scoping reviews and evidence mapping,[20] we will present the results through tables and figures, e.g. a map pointing out where studies have been conducted and summarizing key characteristics. As needed, we will consider other figures such as a matrix evidence.[20]

Dissemination: patient and public involvement

We plan to report our findings in a scientific publication. In addition, an depending on available resources, we aim to produce a website (or implement in an existing website) in which the findings and summarized reports can be easily accessed. Furthermore, we aim to host the machine learning models so that researchers, policymakers and the general public can readily access them. Where the machine learning models are open access or can be accessed through the original reports, these will be hosted on the website or a link to the original source will be provided; conversely, where machine learning models are not open access, we will contact the study authors and ask for the model to be hosted in our website or for a link to their machine learning model. This dissemination plan aims to increase visibility of machine learning research in LMICs and to increase the use of available models, thereby encouraging further research to improve health outcomes.

For peer review only - http://bmjopen.bmj.com/site/about/guidelines.xhtml

AUTHOR STATEMENT

RMC-L conceived the idea and drafted the manuscript. JP-S, TP and RA provided advice to improve the research question and LTC to improve the protocol. JJM and RA edited and provided insights to improve the protocol. All authors approved the submitted version.

For peer review only

For peer review only - http://bmjopen.bmj.com/site/about/guidelines.xhtml

REFERENCES

1. Davies, S.C. “Annual Report of the Chief Medical Officer, 2018 Health 2040 – Better Health Within Reach”: Department of Health and Social Care (2018).

2. Rebala G, Ravi A, Churiwala S. An Introduction to Machine Learning: Springer International Publishing; 2019.

3. Panch T, Pearson-Stuttard J, Greaves F, Atun R. Artificial intelligence: opportunities and risks for public health. *The Lancet Digital Health* 2019; **1**(1): e13-e4.

4. Wahl B, Cossy-Gantner A, Germann S, Schwalbe NR. Artificial intelligence (AI) and global health: how can AI contribute to health in resource-poor settings? *BMJ global health* 2018; **3**(4): e000798.

5. de Filippis R, Carbone EA, Gaetano R, et al. Machine learning techniques in a structural and functional MRI diagnostic approach in schizophrenia: a systematic review. *Neuropsychiatric disease and treatment* 2019; **15**: 1605-27.

6. Bradley A, van der Meer R, McKay C. Personalized Pancreatic Cancer Management: A Systematic Review of How Machine Learning Is Supporting Decision-making. *Pancreas* 2019; **48**(5): 598-604.

7. Shung D, Simonov M, Gentry M, Au B, Laine L. Machine Learning to Predict Outcomes in Patients with Acute Gastrointestinal Bleeding: A Systematic Review. *Digestive diseases and sciences* 2019; **64**(8): 2078-87.

8. Shamseer L, Moher D, Clarke M, et al. Preferred reporting items for systematic review and meta-analysis protocols (PRISMA-P) 2015: elaboration and explanation. *BMJ (Clinical research ed)* 2015; **350**: g7647.

9. Levac D, Colquhoun H, O'Brien KK. Scoping studies: advancing the methodology. *Implementation science : IS* 2010; **5**: 69.

10. Tricco AC, Lillie E, Zarin W, et al. PRISMA Extension for Scoping Reviews (PRISMA-ScR): Checklist and Explanation. *Annals of internal medicine* 2018; **169**(7): 467-73.

For peer review only - http://bmjopen.bmj.com/site/about/guidelines.xhtml

11. The World Bank. World Bank Country and Lending Groups. URL: https://datahelpdesk.worldbank.org/knowledgebase/articles/906519-world-bank-country-and-lending-groups.

12. Burkov A. The Hundred-page Machine Learning Book: Andriy Burkov; 2019.

13. Hu L, Bell D, Antani S, et al. An Observational Study of Deep Learning and Automated Evaluation of Cervical Images for Cancer Screening. *Journal of the National Cancer Institute* 2019.

14. Lorenzoni G, Bressan S, Lanera C, Azzolina D, Da Dalt L, Gregori D. Analysis of Unstructured Text-Based Data Using Machine Learning Techniques: The Case of Pediatric Emergency Department Records in Nicaragua. *Medical care research and review : MCRR* 2019: 1077558719844123.

15. Correa M, Zimic M, Barrientos F, et al. Automatic classification of pediatric pneumonia based on lung ultrasound pattern recognition. *PloS one* 2018; **13**(12): e0206410.

16. Dias RD, Gupta A, Yule SJ. Using Machine Learning to Assess Physician Competence: A Systematic Review. *Academic medicine : journal of the Association of American Medical Colleges* 2019; **94**(3): 427-39.

17. Patil S, Habib Awan K, Arakeri G, et al. Machine learning and its potential applications to the genomic study of head and neck cancer-A systematic review. *Journal of oral pathology & medicine : official publication of the International Association of Oral Pathologists and the American Academy of Oral Pathology* 2019.

18. Wu J, Zhao Y. Machine learning technology in the application of genome analysis: A systematic review. *Gene* 2019; **705**: 149-56.

19. Ouzzani M, Hammady H, Fedorowicz Z, Elmagarmid A. Rayyan-a web and mobile app for systematic reviews. *Systematic reviews* 2016; **5**(1): 210.

20. Evidence and gap maps: a comparison of different approaches. Oslo, Norway: The Campbell Collaboration. Retrieved from: www.campbellcollaboration.org/ DOI: https://doi.org/10.4073/cmdp.2018.2.

For peer review only - http://bmjopen.bmj.com/site/about/guidelines.xhtml

For peer review only

TABLES

Table 1: Overall search terms

1	artificial intelligence.mp.
2	exp Artificial Intelligence/
3	machine learning.mp.
4	exp machine learning/
5	deep learning.mp.
6	unsupervised machine learning.mp.
7	supervised machine learning.mp.
8	computational Intelligence.mp.
9	predictive analytic*.mp.
10	support vector machine.mp.
11	support vector regression.mp.
12	decision tree*.mp.
13	random forest.mp.
14	neural network*.mp.
15	exp Neural Networks/
16	bayesian network*.mp.
17	artificial neural network*.mp.
18	convolutional neural network*.mp.
19	computer vision systems.mp.
20	exp Image Processing, Computer-Assisted/
21	natural language processesing.mp.
22	1 or 2 or 3 …or 21
23	(("Afghanistan") or ("Benin") or ("Burkina Faso") or ("Burundi") or ("Central African Republic") or ("Chad") or ("Comoros") or ("Democratic Republic of the Congo") or ("Eritrea") or ("Ethiopia") or ("Gambia") or ("Guinea") or ("Guinea-Bissau") or ("Haiti") or ("Democratic People's Republic of Korea") or ("Liberia") or ("Madagascar") or ("Malawi") or ("Mali") or ("Mozambique") or ("Nepal") or ("Niger") or ("Rwanda") or ("Senegal") or ("Sierra Leone") or ("Somalia") or ("South Sudan") or ("Tanzania") or ("Togo") or ("Uganda") or ("Zimbabwe") or ("Armenia") or ("Bangladesh") or ("Bhutan") or ("Bolivia") or ("Cape Verde") or ("Cambodia") or ("Cameroon") or ("Congo") or ("Cote d'Ivoire") or ("Djibouti") or ("Egypt") or ("El Salvador") or ("Ghana") or ("Guatemala") or ("Honduras") or ("India") or ("Indonesia") or ("Kenya") or ("Micronesia") or ("Kosovo") or ("Kyrgyzstan") or ("Laos") or ("Lesotho") or ("Mauritania") or ("Moldova") or ("Mongolia") or ("Morocco") or ("Myanmar") or ("Nicaragua") or ("Nigeria") or ("Pakistan") or ("Papua New Guinea") or ("Philippines") or ("Samoa") or ("Atlantic Islands") or ("Melanesia") or ("Sri Lanka") or ("Sudan") or ("Swaziland") or ("Syria") or ("Tajikistan") or ("Timor-Leste") or ("Tonga") or ("Tunisia") or ("Ukraine") or ("Uzbekistan") or ("Vanuatu") or ("Vietnam") or ("Middle East") or ("Yemen") or ("Zambia") or ("Albania") or ("Algeria") or ("American Samoa") or ("Angola") or ("Argentina") or ("Azerbaijan") or ("Republic of Belarus") or ("Belize") or ("Bosnia and Herzegovina") or ("Botswana") or ("Brazil") or ("Bulgaria") or ("China") or ("Colombia") or ("Costa Rica") or ("Cuba") or ("Dominica") or ("Dominican Republic") or ("Equatorial Guinea") or ("Ecuador") or ("Fiji") or ("Gabon") or ("Georgia") or ("Grenada") or ("Guyana") or ("Iran") or ("Iraq") or ("Jamaica") or ("Jordan") or ("Kazakhstan") or ("Lebanon") or ("Libya") or ("Macedonia (Republic)") or ("Malaysia") or ("Indian Ocean Islands") or ("Mauritius") or ("Mexico") or ("Montenegro") or ("Namibia") or ("Palau") or

For peer review only - http://bmjopen.bmj.com/site/about/guidelines.xhtml

	("Panama") or ("Paraguay") or ("Peru") or ("Romania") or ("Russia") or ("Serbia") or ("South Africa") or ("Saint Lucia") or ("Saint Vincent and the Grenadines") or ("Suriname") or ("Thailand") or ("Turkey") or ("Turkmenistan") or ("Venezuela") or (developing countr) or (low-income countr*) or (middle-income countr*) or (low-middle income countr*) or (upper-middle income countr*))
24	22 and 23
25	exp animals/ not humans.sh.
26	24 not 25
27	Remove duplicates from 26

For peer review only

For peer review only - http://bmjopen.bmj.com/site/about/guidelines.xhtml

FIGURES

Figure 1: Algorithm to filter search results

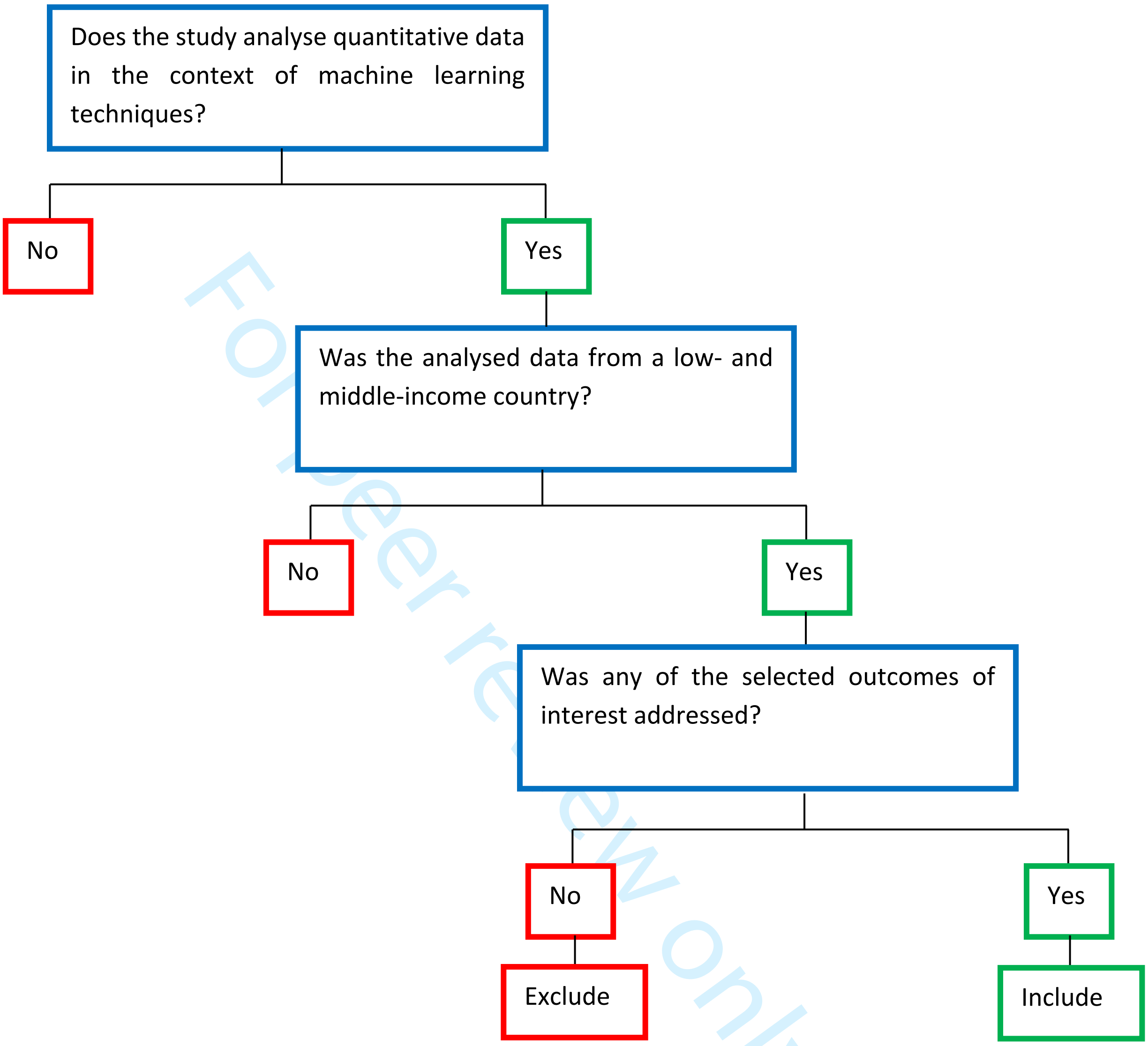

For peer review only - http://bmjopen.bmj.com/site/about/guidelines.xhtml

SUPPLEMENTARY MATERIAL

A. *S1: PRISMA-P checklist*

PRISMA-P (Preferred Reporting Items for Systematic review and Meta-Analysis Protocols) 2015 checklist: recommended items to address in a systematic review protocol

Section and topic	Item No	Checklist item	Page	Paragraph
**ADMINISTRATIVE INFORMATION**				
Title:				
Identification	1a	Identify the report as a protocol of a systematic review	1	1
Update	1b	If the protocol is for an update of a previous systematic review, identify as such	NA	NA
Registration	2	If registered, provide the name of the registry (such as PROSPERO) and registration number	NA	NA
Authors:				
Contact	3a	Provide name, institutional affiliation, e-mail address of all protocol authors; provide physical mailing address of corresponding author	1	1
Contributions	3b	Describe contributions of protocol authors and identify the guarantor of the review	2	3
Amendments	4	If the protocol represents an amendment of a previously completed or published protocol, identify as such and list changes; otherwise, state plan for documenting important protocol amendments	NA	NA
Support:				
Sources	5a	Indicate sources of financial or other support for the review	2	2
Sponsor	5b	Provide name for the review funder and/or sponsor	2	2
Role of sponsor or funder	5c	Describe roles of funder(s), sponsor(s), and/or institution(s), if any, in developing the protocol	2	2
**INTRODUCTION**				
Rationale	6	Describe the rationale for the review in the context of what is already known	5	2

For peer review only

Objectives	7	Provide an explicit statement of the question(s) the review will address with reference to participants, interventions, comparators, and outcomes (PICO)	5	3
**METHODS**				
Eligibility criteria	8	Specify the study characteristics (such as PICO, study design, setting, time frame) and report characteristics (such as years considered, language, publication status) to be used as criteria for eligibility for the review	6	6
Information sources	9	Describe all intended information sources (such as electronic databases, contact with study authors, trial registers or other grey literature sources) with planned dates of coverage	8	2
Search strategy	10	Present draft of search strategy to be used for at least one electronic database, including planned limits, such that it could be repeated	8	3
Study records:				
Data management	11a	Describe the mechanism(s) that will be used to manage records and data throughout the review	8	4
Selection process	11b	State the process that will be used for selecting studies (such as two independent reviewers) through each phase of the review (that is, screening, eligibility and inclusion in meta-analysis)	8	5
Data collection process	11c	Describe planned method of extracting data from reports (such as piloting forms, done independently, in duplicate), any processes for obtaining and confirming data from investigators	8	5
Data items	12	List and define all variables for which data will be sought (such as PICO items, funding sources), any pre-planned data assumptions and simplifications	NA	NA
Outcomes and prioritization	13	List and define all outcomes for which data will be sought, including prioritization of main and additional outcomes, with rationale	7	4
Risk of bias in individual studies	14	Describe anticipated methods for assessing risk of bias of individual studies, including whether this will be done at the outcome or study level, or both; state how this information will be used in data synthesis	9	2
Data synthesis	15a	Describe criteria under which study data will be quantitatively synthesised	9	3
	15b	If data are appropriate for quantitative synthesis, describe planned summary measures, methods of handling data and methods of combining data from studies, including any planned exploration of consistency (such as I^2, Kendall's τ)	NA	NA
	15c	Describe any proposed additional analyses (such as sensitivity or subgroup analyses, meta-regression)	NA	NA
	15d	If quantitative synthesis is not appropriate, describe the type of summary planned	NA	NA

For peer review only

Meta-bias(es)	16	Specify any planned assessment of meta-bias(es) (such as publication bias across studies, selective reporting within studies)	NA	NA
Confidence in cumulative evidence	17	Describe how the strength of the body of evidence will be assessed (such as GRADE)	NA	NA

From: Shamseer L, Moher D, Clarke M, Ghersi D, Liberati A, Petticrew M, Shekelle P, Stewart L, PRISMA-P Group. Preferred reporting items for systematic review and meta-analysis protocols (PRISMA-P) 2015: elaboration and explanation. BMJ. 2015 Jan 2;349(jan02 1):g7647

For peer review only

BMJ Open

Machine learning health-related applications in low- and middle-income countries: A scoping review protocol

Journal:	*BMJ Open*
Manuscript ID	bmjopen-2019-035983.R1
Article Type:	Protocol
Date Submitted by the Author:	11-Mar-2020
Complete List of Authors:	Carrillo-Larco, Rodrigo; Universidad Peruana Cayetano Heredia, CRONICAS Center of Excellence; CRONICAS Center of Excellence in Chronic Diseases<br>Tudor Car, Lorainne; Lee Kong Chian School of Medicine, Family Medicine and Primary Care<br>Pearson-Stuttard, Jonathan; Imperial College London, PCPH<br>Panch, Trishan; Wellframe Inc.<br>Miranda, J. Jaime; Universidad Peruana Cayetano Heredia, CRONICAS Centre of Excellence in Chronic Diseases<br>Atun, Rifat; Harvard University, Harvard School of Public Health
**Primary Subject Heading**:	Global health
Secondary Subject Heading:	Diagnostics, Epidemiology
Keywords:	EPIDEMIOLOGY, BIOTECHNOLOGY & BIOINFORMATICS, Health informatics < BIOTECHNOLOGY & BIOINFORMATICS, World Wide Web technology < BIOTECHNOLOGY & BIOINFORMATICS

SCHOLARONE™
Manuscripts

I, the Submitting Author has the right to grant and does grant on behalf of all authors of the Work (as defined in the below author licence), an exclusive licence and/or a non-exclusive licence for contributions from authors who are: i) UK Crown employees; ii) where BMJ has agreed a CC-BY licence shall apply, and/or iii) in accordance with the terms applicable for US Federal Government officers or employees acting as part of their official duties; on a worldwide, perpetual, irrevocable, royalty-free basis to BMJ Publishing Group Ltd ("BMJ") its licensees and where the relevant Journal is co-owned by BMJ to the co-owners of the Journal, to publish the Work in this journal and any other BMJ products and to exploit all rights, as set out in our licence.

The Submitting Author accepts and understands that any supply made under these terms is made by BMJ to the Submitting Author unless you are acting as an employee on behalf of your employer or a postgraduate student of an affiliated institution which is paying any applicable article publishing charge ("APC") for Open Access articles. Where the Submitting Author wishes to make the Work available on an Open Access basis (and intends to pay the relevant APC), the terms of reuse of such Open Access shall be governed by a Creative Commons licence – details of these licences and which Creative Commons licence will apply to this Work are set out in our licence referred to above.

Other than as permitted in any relevant BMJ Author's Self Archiving Policies, I confirm this Work has not been accepted for publication elsewhere, is not being considered for publication elsewhere and does not duplicate material already published. I confirm all authors consent to publication of this Work and authorise the granting of this licence.

For peer review only

Machine learning health-related applications in low- and middle-income countries: A scoping review protocol

Rodrigo M Carrillo-Larco (r.carrillo-larco@imperial.ac.uk) [1,2]

Lorainne Tudor Car (lorainne.tudor.car@ntu.edu.sg) [3,4]

Jonathan Pearson-Stuttard (j.pearson-stuttard@imperial.ac.uk) [5]

Trishan Panch (trishan@wellframe.com) [6]

J Jaime Miranda (jaime.miranda@upch.pe) [2,7]

Rifat Atun (ratun@hsph.harvard.edu) [8]

1. Department of Epidemiology and Biostatistics, School of Public Health, Imperial College London, London, UK.
2. CRONICAS Centre of Excellence in Chronic Diseases, Universidad Peruana Cayetano Heredia, Lima, Peru.
3. Family Medicine and Primary Care, Lee Kong Chian School of Medicine, Nanyang Technological University, Singapore.
4. Department of Primary Care and Public Health, School of Public Health, Imperial College London, London, UK.
5. Department of Epidemiology and Biostatistics and MRC-PHE Centre for Environment and Health, School of Public Health, Imperial College London, London, UK.
6. Wellframe Inc., Boston, Massachusetts, USA.
7. Facultad de Medicina "Alberto Hurtado", Universidad Peruana Cayetano Heredia, Lima, Peru.
8. Harvard T.H Chan, School of Public Health, and Harvard Medical School, Harvard University, Cambridge, Massachusetts, USA.

For peer review only - http://bmjopen.bmj.com/site/about/guidelines.xhtml

For peer review only

Corresponding author:

Rodrigo M. Carrillo-Larco, MD

Department of Epidemiology and Biostatistics

School of Public Health

Imperial College London, London, UK

r.carrillo-larco@imperial.ac.uk

Key words: Artificial intelligence; Algorithms; Developing countries

Word count: 1,540

For peer review only

ABSTRACT

Introduction: Machine learning (ML) has been used in bio-medical research, and recently in clinical and public health research. However, much of the available evidence comes from high-income countries where different health profiles challenges the application of this research to low- and middle-income countries (LMICs). It is largely unknown what ML applications are available for LMICs that can support and advance clinical medicine and public health. We aim to address this gap by conducting a scoping review of health-related ML applications in LMICs.

Methods and analysis: This scoping review will follow the methodology proposed by Levac *et al*. The search strategy is informed by recent systematic reviews of ML health-related applications. We will search Embase, Medline and Global Health (through OVID), Cochrane and Google Scholar. Titles and abstracts will be screened by two reviewers independently; selected reports will be studied by two reviewers independently. Reports will be included if they are primary research where data have been analysed, ML techniques have been used on data from LMICs, and they aimed to improve health-related outcomes. We will synthesize the information following evidence mapping recommendations.

Ethics and dissemination: The review will provide a comprehensive list of health-related ML applications in LMICs. The results will be disseminated through scientific publications. We also plan to launch a website where ML models can be hosted so that researchers, policymakers and the general public can readily access them.

For peer review only

For peer review only - http://bmjopen.bmj.com/site/about/guidelines.xhtml

STRENGTHS AND LIMITATIONS OF THIS STUDY

- This protocol outlines a comprehensive scoping review to map machine learning health-related applications for low- and middle-income countries, an enterprise not conducted before.
- The search strategy is informed by a solid framework, the search will be conducted in several information sources, and the screening/review will be performed by two reviewers independently.
- As part of the dissemination plan, we aim to launch a website where the results and original machine learning models can be readily accessed, fostering the using and further research of machine learning applications in low- and middle-income countries.
- We will not search abstracts or conference proceedings specialized in machine learning, focusing on information sources where health-related outcomes are most likely to be found.

For peer review only

For peer review only - http://bmjopen.bmj.com/site/about/guidelines.xhtml

INTRODUCTION

Machine learning refers to the process through which computers, models or algorithms, learn and improve from data and processes, rather than from specific programmed instructions.[1,2] Machine learning can be used in tasks such as classification (e.g., whether a tumour is benign or malign based on patterns or characteristics), clustering (e.g., group patients with similar profiles for targeted prevention or treatment interventions) or prediction (e.g., forecast propensity to risk or probability of outcome of a disease following interventions).[2] Of note, there could be overlap across different tasks.

Machine learning is widely used in bio-medical sciences, and more recently in clinical and public health research.[3,4] Systematic reviews on health-related applications of machine learning have explored questions such as the accuracy of machine learning for diagnosis or outcome prediction,[5-7] but most of the research studies included in these reviews have come from high-income countries and the findings may not apply to low-income and middle-income countries (LMICs) because the variability in access to healthcare and difference in the disease burden. It is largely unknown what machine learning applications are available for LMICs that can support and advance clinical medicine and public health.

Our aim was to address this gap in the evidence by conducting a scoping review of health-related applications of machine learning in LMICs to synthesise published evidence and to garner lessons to inform further research and policy development. The review will provide the first comprehensive list of health-related machine learning applications in LMICs.

METHODS

Overview

This is a scoping review of the published scientific literature. This protocol adheres to the PRISMA-P guidelines (supplementary material S1),[8] and the methodology follows the procedures suggested by Levac et al.[9] The final publication of this work will adhere to the PRISMA-ScR recommendations.[10]

For peer review only - http://bmjopen.bmj.com/site/about/guidelines.xhtml

PRISMA-P is a standard method to report review protocols; similarly, PRSIMA-ScR is a well-known instrument to guide the reporting of scoping reviews. The methodology proposed by Levac and colleagues is useful for scoping reviews that aim to generate a broad picture of the available evidence on a subject.

Understanding that a key feature of scoping reviews is a broad research question, we aim to answer: *what have been the health-related applications of machine learning techniques in LMICs?* This review will summarize scientific evidence on research that have used data from LMICs for machine learning applications in clinical medicine (e.g., risk prediction for clinical decisions) and public health (e.g., vector control in an endemic area) to provide solutions to health problems in LMICs.

Definitions

For this work we will follow these definitions:

- LMICs: classification by the World Bank country income grouping (see search strategy in Table 1 for a list of countries).[11]
- Machine learning: analytical techniques through which computers learn directly from data, examples and experiences, rather than from a pre-programmed rule.[1,2]
- Machine learning techniques: analytical methods within the machine learning remit, i.e. where machines have learnt from data or processes through machine learning techniques or algorithms. These include, but are not limited to:[2,12] support vector machine, support vector regression, decision trees, random forest, neural network, Bayesian network, artificial neural network, computer vision systems, computer-assisted image processing and natural language processing.

Eligibility criteria

We will include scientific evidence that meets the following inclusion criteria (Figure 1):

For peer review only - http://bmjopen.bmj.com/site/about/guidelines.xhtml

Primary research studies.

We will include experimental and observational studies that have used and reported quantitative data. We will also screen relevant systematic reviews or narrative reviews for eligible primary studies and include them in our review if relevant. We will not include qualitative studies, opinions, conference abstracts, letters, editorials or any other scientific work in which data have not been actively analysed within a machine learning framework.

Machine learning research should have used data from LMICs, i.e. machine learning research that used solely data from LMICs.

We will focus on machine learning research based on data from LMICs applied to LMICs. We will not include machine learning research that used data from high-income countries even though it could have been applied to a LMIC, and neither studies using LMICs data applied to high-income countries. This scoping review focuses on the applications of machine learning techniques in LMICs; these applications should have been developed using LMICs data, because prediction models and other algorithms work better, i.e. have better accuracy, in the populations – data – for which they were developed. Conversely, when these models are applied to other populations, settings or data, they need some modification (e.g., re-calibration). We will also exclude studies that have used LMIC data in a consortium or data pooling group if the model or results cannot be separated for the LMIC alone; in other words, we will exclude a report if this used LMIC data in aggregate with data from high-income countries, but the application cannot be separated for the LMIC alone.

Models developed in sites other than LMICs may not work correctly in these countries. For example, projects with digital imaging from high-income countries may reflect a different scenario; i.e., images from streets in LMICs may depict objects or features not found in high-income countries. Another example could be projects for sound/noise classification. Those from high-income countries may not identify the variety of noises usually available in LIMCs (e.g., loud cars or indiscriminate use of car

For peer review only - http://bmjopen.bmj.com/site/about/guidelines.xhtml

horns). Finally, LMICs still have sizable rural areas with large populations. Extrapolating models built for highly urbanized cities may not be adequate for rural sites.

Examples of studies of interest include: i) development of a 'deep learning-based visual evaluation algorithm' to early identify cervical cancer signs based on data from women in Costa Rica.[13] ii) Classification of free-text (random forest) in emergency department records from nine hospitals in Nicaragua.[14] iii) Automatic classification (neural networks) of paediatric pneumonia based on ultrasound records from children in Peru.[15] During the selection stages.

Distinguishing between machine learning applications and more conventional statistical methods could be challenging because in some cases the definitions are unclear; for instance, regression analysis. Nonetheless, from the context of the scientific paper, from the aims or overall methodological approach, it is possible to reckon whether a study uses machine learning techniques versus more conventional statistical methods. If needed, we would reach out to authors for further information.

The outcome of the study/analysis was to improve a health-related outcome.

The *primary outcome* of the selected studies should have sought improvements in the following health-related endpoints along the care cascade: diagnosis, treatment, control, survival, complications and mortality. These outcomes have been selected because of their relevance in healthcare, clinical medicine and public health. As *secondary outcome* we will also include studies that have reported endpoints related to cost, efficiency and productivity in the healthcare process.

Information sources

We will conduct the search in three databases through Ovid: Embase, Medline and Global Health; in addition, we will search Cochrane and Google Scholar (first 10 pages). Besides Google Scholar, no other grey literature source will be searched. These sources will be used without language or time/year restrictions. We have a very diverse team covering many languages and we will tap into our

For peer review only - http://bmjopen.bmj.com/site/about/guidelines.xhtml

For peer review only

networks at our institutions in case we come across a study in a language that the authors do not speak.

Search strategy

Based on recent systematic reviews of machine learning applications on health-related endpoints: outcome prediction in gastrointestinal bleeding,[7] assessment of physicians knowledge,[16] applications on genomic data to predict outcomes in cancer patients,[17] and available machine learning algorithms to improve genomic data analysis,[18] we developed our search strategy (Table 1). We will screen the references of the included reports for any other relevant studies.

Study records

Results will be downloaded into EndNote where duplicates will be omitted, and a second cleaning of duplicated results will be conducted using the online tool Rayyan.[19] Titles and abstracts will be reviewed by two independent researchers following the above detailed selection criteria (Figure 1); discrepancies will be solved by consensus or by a third party. Selected titles will be sought in full-text, which will be assessed by two independent reviewers following the same selection criteria (Figure 1). Again, in case of conflicts, these will be solved by consensus or by a third party.

Data collection

The reviewers will decide on a list of items that will be extracted to successfully answer the research question. These items will be implemented in an Excel spreadsheet before data extraction and will not be modified afterwards. Information of interest includes: country of origin; analytical approach; type of data used; data source; model performance; outcome of interest; number of observations; and whether the model is available for independent use or reproduction. Data from the selected reports will be extracted by two researchers independently; if there were discrepancies, these will be solved by consensus among them or by a third party.

For peer review only

Risk of bias in individual studies

Because this is a scoping review aiming to summarise available evidence to identify research gaps and potential uses of machine learning to improve health-related outcomes in LMICs, no risk of bias of individual studies is planned.

Data synthesis

We anticipate a large heterogeneity of selected reports, both in terms of methodology and outcomes, as well as target population and data sources. Therefore, no meta-analysis is planned and only a qualitative summary will be conducted. Following current recommendations for scoping reviews and evidence mapping,[20] we will present the results through tables and figures, e.g. a map pointing out where studies have been conducted and summarizing key characteristics. As needed, we will consider other figures such as a matrix evidence.[20]

Dissemination: patient and public involvement

We plan to report our findings in a scientific publication. In addition, and depending on available resources, we aim to produce a website (or implement in an existing website) in which the findings and summarized reports can be easily accessed. Furthermore, we aim to host the machine learning models so that researchers, policymakers and the general public can readily access them. Where the machine learning models are open access or can be accessed through the original reports, these will be hosted on the website or a link to the original source will be provided; conversely, where machine learning models are not open access, we will contact the study authors and ask for the model to be hosted in our website or for a link to their machine learning model. This dissemination plan aims to increase visibility of machine learning research in LMICs and to increase the use of available models, thereby encouraging further research to improve health outcomes. We will engage with the communication office in our universities to promote this website through relevant channels including but not limited to social media, newsletters and institutional websites.

For peer review only

CONTRIBUTION STATEMENT

RMC-L conceived the idea and drafted the manuscript. JP-S, TP and RA provided advice to improve the research question and LTC to improve the protocol. JJM and RA edited and provided insights to improve the protocol. All authors approved the submitted version.

COMPETING INTERESTS

None to declare.

FUNDING

RMC-L has been supported by a Strategic Award, Wellcome Trust-Imperial College Centre for Global Health Research (100693/Z/12/Z), and Imperial College London Wellcome Trust Institutional Strategic Support Fund [Global Health Clinical Research Training Fellowship] (294834/Z/16/Z ISSF ICL). RMC-L is supported by a Wellcome Trust International Training Fellowship (214185/Z/18/Z). The funders had no role in this work and decision to submit for publication.

DATA SHARING STATEMENT

Not applicable

For peer review only

REFERENCES

1. Davies, S.C. "Annual Report of the Chief Medical Officer, 2018 Health 2040 – Better Health Within Reach": Department of Health and Social Care (2018).

2. Rebala G, Ravi A, Churiwala S. An Introduction to Machine Learning: Springer International Publishing; 2019.

3. Panch T, Pearson-Stuttard J, Greaves F, Atun R. Artificial intelligence: opportunities and risks for public health. *The Lancet Digital Health* 2019; **1**(1): e13-e4.

4. Wahl B, Cossy-Gantner A, Germann S, Schwalbe NR. Artificial intelligence (AI) and global health: how can AI contribute to health in resource-poor settings? *BMJ global health* 2018; **3**(4): e000798.

5. de Filippis R, Carbone EA, Gaetano R, et al. Machine learning techniques in a structural and functional MRI diagnostic approach in schizophrenia: a systematic review. *Neuropsychiatric disease and treatment* 2019; **15**: 1605-27.

6. Bradley A, van der Meer R, McKay C. Personalized Pancreatic Cancer Management: A Systematic Review of How Machine Learning Is Supporting Decision-making. *Pancreas* 2019; **48**(5): 598-604.

7. Shung D, Simonov M, Gentry M, Au B, Laine L. Machine Learning to Predict Outcomes in Patients with Acute Gastrointestinal Bleeding: A Systematic Review. *Digestive diseases and sciences* 2019; **64**(8): 2078-87.

8. Shamseer L, Moher D, Clarke M, et al. Preferred reporting items for systematic review and meta-analysis protocols (PRISMA-P) 2015: elaboration and explanation. *BMJ (Clinical research ed)* 2015; **350**: g7647.

9. Levac D, Colquhoun H, O'Brien KK. Scoping studies: advancing the methodology. *Implementation science : IS* 2010; **5**: 69.

10. Tricco AC, Lillie E, Zarin W, et al. PRISMA Extension for Scoping Reviews (PRISMA-ScR): Checklist and Explanation. *Annals of internal medicine* 2018; **169**(7): 467-73.

For peer review only

For peer review only - http://bmjopen.bmj.com/site/about/guidelines.xhtml

11. The World Bank. World Bank Country and Lending Groups. URL: https://datahelpdesk.worldbank.org/knowledgebase/articles/906519-world-bank-country-and-lending-groups.

12. Burkov A. The Hundred-page Machine Learning Book: Andriy Burkov; 2019.

13. Hu L, Bell D, Antani S, et al. An Observational Study of Deep Learning and Automated Evaluation of Cervical Images for Cancer Screening. *Journal of the National Cancer Institute* 2019.

14. Lorenzoni G, Bressan S, Lanera C, Azzolina D, Da Dalt L, Gregori D. Analysis of Unstructured Text-Based Data Using Machine Learning Techniques: The Case of Pediatric Emergency Department Records in Nicaragua. *Medical care research and review : MCRR* 2019: 1077558719844123.

15. Correa M, Zimic M, Barrientos F, et al. Automatic classification of pediatric pneumonia based on lung ultrasound pattern recognition. *PloS one* 2018; **13**(12): e0206410.

16. Dias RD, Gupta A, Yule SJ. Using Machine Learning to Assess Physician Competence: A Systematic Review. *Academic medicine : journal of the Association of American Medical Colleges* 2019; **94**(3): 427-39.

17. Patil S, Habib Awan K, Arakeri G, et al. Machine learning and its potential applications to the genomic study of head and neck cancer-A systematic review. *Journal of oral pathology & medicine : official publication of the International Association of Oral Pathologists and the American Academy of Oral Pathology* 2019.

18. Wu J, Zhao Y. Machine learning technology in the application of genome analysis: A systematic review. *Gene* 2019; **705**: 149-56.

19. Ouzzani M, Hammady H, Fedorowicz Z, Elmagarmid A. Rayyan-a web and mobile app for systematic reviews. *Systematic reviews* 2016; **5**(1): 210.

20. Evidence and gap maps: a comparison of different approaches. Oslo, Norway: The Campbell Collaboration. Retrieved from: www.campbellcollaboration.org/ DOI: https://doi.org/10.4073/cmdp.2018.2.

For peer review only - http://bmjopen.bmj.com/site/about/guidelines.xhtml

TABLES

Table 1: Overall search terms

1	artificial intelligence.mp.
2	exp Artificial Intelligence/
3	machine learning.mp.
4	exp machine learning/
5	deep learning.mp.
6	unsupervised machine learning.mp.
7	supervised machine learning.mp.
8	computational Intelligence.mp.
9	predictive analytic*.mp.
10	support vector machine.mp.
11	support vector regression.mp.
12	decision tree*.mp.
13	random forest.mp.
14	neural network*.mp.
15	exp Neural Networks/
16	bayesian network*.mp.
17	artificial neural network*.mp.
18	convolutional neural network*.mp.
19	computer vision systems.mp.
20	exp Image Processing, Computer-Assisted/
21	natural language processesing.mp.
22	1 or 2 or 3 …or 21
23	(("Afghanistan") or ("Benin") or ("Burkina Faso") or ("Burundi") or ("Central African Republic") or ("Chad") or ("Comoros") or ("Democratic Republic of the Congo") or ("Eritrea") or ("Ethiopia") or ("Gambia") or ("Guinea") or ("Guinea-Bissau") or ("Haiti") or ("Democratic People's Republic of Korea") or ("Liberia") or ("Madagascar") or ("Malawi") or ("Mali") or ("Mozambique") or ("Nepal") or ("Niger") or ("Rwanda") or ("Senegal") or ("Sierra Leone") or ("Somalia") or ("South Sudan") or ("Tanzania") or ("Togo") or ("Uganda") or ("Zimbabwe") or ("Armenia") or ("Bangladesh") or ("Bhutan") or ("Bolivia") or ("Cape Verde") or ("Cambodia") or ("Cameroon") or ("Congo") or ("Cote d'Ivoire") or ("Djibouti") or ("Egypt") or ("El Salvador") or ("Ghana") or ("Guatemala") or ("Honduras") or ("India") or ("Indonesia") or ("Kenya") or ("Micronesia") or ("Kosovo") or ("Kyrgyzstan") or ("Laos") or ("Lesotho") or ("Mauritania") or ("Moldova") or ("Mongolia") or ("Morocco") or ("Myanmar") or ("Nicaragua") or ("Nigeria") or ("Pakistan") or ("Papua New Guinea") or ("Philippines") or ("Samoa") or ("Atlantic Islands") or ("Melanesia") or ("Sri Lanka") or ("Sudan") or ("Swaziland") or ("Syria") or ("Tajikistan") or ("Timor-Leste") or ("Tonga") or ("Tunisia") or ("Ukraine") or ("Uzbekistan") or ("Vanuatu") or ("Vietnam") or ("Middle East") or ("Yemen") or ("Zambia") or ("Albania") or ("Algeria") or ("American Samoa") or ("Angola") or ("Argentina") or ("Azerbaijan") or ("Republic of Belarus") or ("Belize") or ("Bosnia and Herzegovina") or ("Botswana") or ("Brazil") or ("Bulgaria") or ("China") or ("Colombia") or ("Costa Rica") or ("Cuba") or ("Dominica") or ("Dominican Republic") or ("Equatorial Guinea") or ("Ecuador") or ("Fiji") or ("Gabon") or ("Georgia") or ("Grenada") or ("Guyana") or ("Iran") or ("Iraq") or ("Jamaica") or ("Jordan") or ("Kazakhstan") or ("Lebanon") or ("Libya") or ("Macedonia (Republic)") or ("Malaysia") or ("Indian Ocean Islands") or ("Mauritius") or ("Mexico") or ("Montenegro") or ("Namibia") or ("Palau") or

For peer review only

	("Panama") or ("Paraguay") or ("Peru") or ("Romania") or ("Russia") or ("Serbia") or ("South Africa") or ("Saint Lucia") or ("Saint Vincent and the Grenadines") or ("Suriname") or ("Thailand") or ("Turkey") or ("Turkmenistan") or ("Venezuela") or (developing countr) or (low-income countr*) or (middle-income countr*) or (low-middle income countr*) or (upper-middle income countr*))
24	22 and 23
25	exp animals/ not humans.sh.
26	24 not 25
27	Remove duplicates from 26

For peer review only

FIGURES

Figure 1: Algorithm to filter search results

For peer review only

For peer review only - http://bmjopen.bmj.com/site/about/guidelines.xhtml

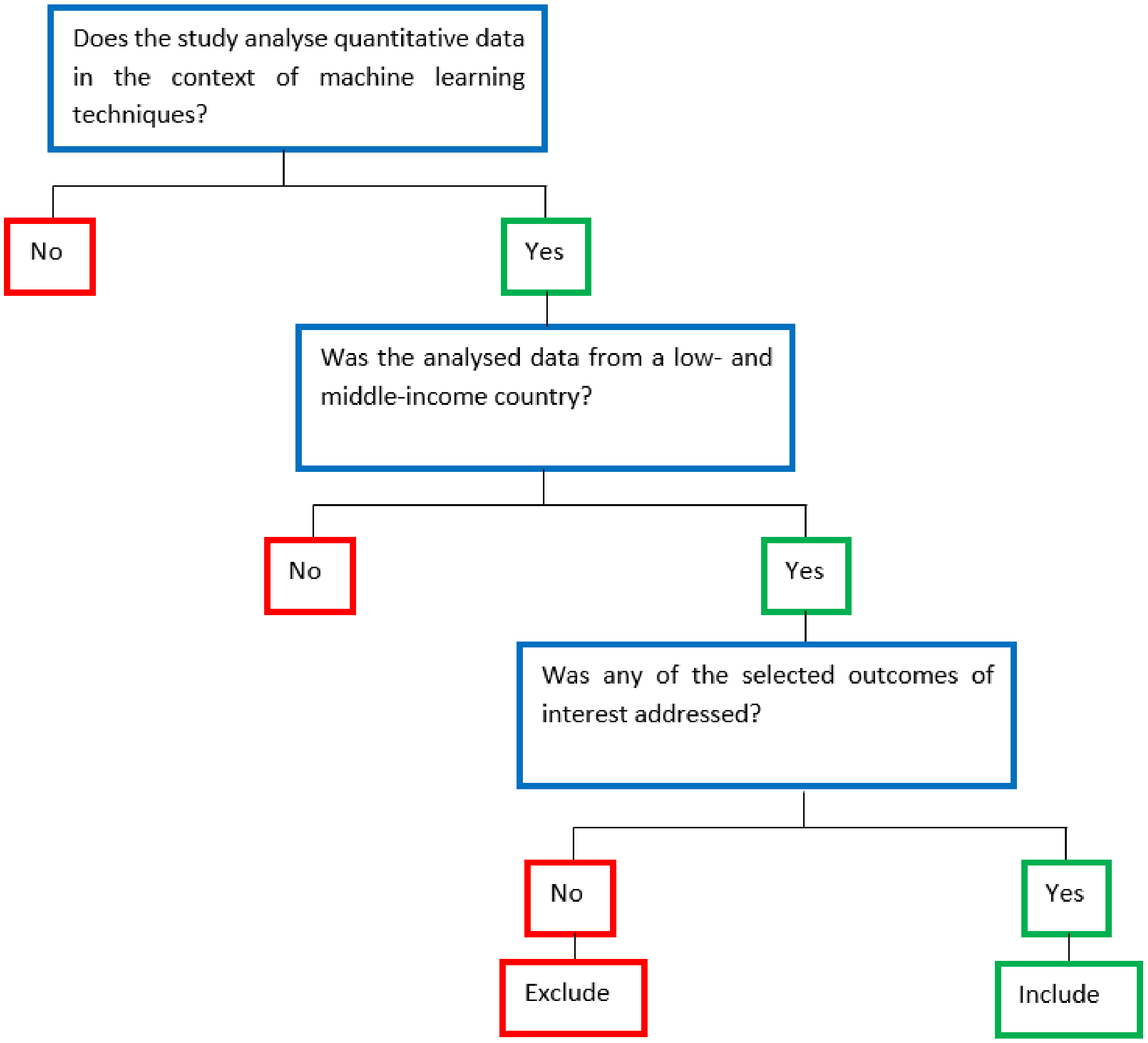
Does the study analyse quantitative data in the context of machine learning techniques?
No
Yes
Was the analysed data from a low- and middle-income country?
No
Yes
Was any of the selected outcomes of interest addressed?
No
Yes
Exclude
Include

w only

SUPPLEMENTARY MATERIAL

*A. **S1: PRISMA-P checklist***

PRISMA-P (Preferred Reporting Items for Systematic review and Meta-Analysis Protocols) 2015 checklist: recommended items to address in a systematic review protocol

Section and topic	Item No	Checklist item	Page	Paragraph
**ADMINISTRATIVE INFORMATION**				
Title:				
Identification	1a	Identify the report as a protocol of a systematic review	1	1
Update	1b	If the protocol is for an update of a previous systematic review, identify as such	NA	NA
Registration	2	If registered, provide the name of the registry (such as PROSPERO) and registration number	NA	NA
Authors:				
Contact	3a	Provide name, institutional affiliation, e-mail address of all protocol authors; provide physical mailing address of corresponding author	1	1
Contributions	3b	Describe contributions of protocol authors and identify the guarantor of the review	2	3
Amendments	4	If the protocol represents an amendment of a previously completed or published protocol, identify as such and list changes; otherwise, state plan for documenting important protocol amendments	NA	NA
Support:				
Sources	5a	Indicate sources of financial or other support for the review	2	2
Sponsor	5b	Provide name for the review funder and/or sponsor	2	2
Role of sponsor or funder	5c	Describe roles of funder(s), sponsor(s), and/or institution(s), if any, in developing the protocol	2	2
**INTRODUCTION**				
Rationale	6	Describe the rationale for the review in the context of what is already known	5	2

For peer review only

Objectives	7	Provide an explicit statement of the question(s) the review will address with reference to participants, interventions, comparators, and outcomes (PICO)	5	3
**METHODS**				
Eligibility criteria	8	Specify the study characteristics (such as PICO, study design, setting, time frame) and report characteristics (such as years considered, language, publication status) to be used as criteria for eligibility for the review	6	6
Information sources	9	Describe all intended information sources (such as electronic databases, contact with study authors, trial registers or other grey literature sources) with planned dates of coverage	8	2
Search strategy	10	Present draft of search strategy to be used for at least one electronic database, including planned limits, such that it could be repeated	8	3
Study records:				
Data management	11a	Describe the mechanism(s) that will be used to manage records and data throughout the review	8	4
Selection process	11b	State the process that will be used for selecting studies (such as two independent reviewers) through each phase of the review (that is, screening, eligibility and inclusion in meta-analysis)	8	5
Data collection process	11c	Describe planned method of extracting data from reports (such as piloting forms, done independently, in duplicate), any processes for obtaining and confirming data from investigators	8	5
Data items	12	List and define all variables for which data will be sought (such as PICO items, funding sources), any pre-planned data assumptions and simplifications	NA	NA
Outcomes and prioritization	13	List and define all outcomes for which data will be sought, including prioritization of main and additional outcomes, with rationale	7	4
Risk of bias in individual studies	14	Describe anticipated methods for assessing risk of bias of individual studies, including whether this will be done at the outcome or study level, or both; state how this information will be used in data synthesis	9	2
Data synthesis	15a	Describe criteria under which study data will be quantitatively synthesised	9	3
	15b	If data are appropriate for quantitative synthesis, describe planned summary measures, methods of handling data and methods of combining data from studies, including any planned exploration of consistency (such as I^2, Kendall's τ)	NA	NA
	15c	Describe any proposed additional analyses (such as sensitivity or subgroup analyses, meta-regression)	NA	NA
	15d	If quantitative synthesis is not appropriate, describe the type of summary planned	NA	NA

For peer review only

Meta-bias(es)	16	Specify any planned assessment of meta-bias(es) (such as publication bias across studies, selective reporting within studies)	NA	NA
Confidence in cumulative evidence	17	Describe how the strength of the body of evidence will be assessed (such as GRADE)	NA	NA

From: Shamseer L, Moher D, Clarke M, Ghersi D, Liberati A, Petticrew M, Shekelle P, Stewart L, PRISMA-P Group. Preferred reporting items for systematic review and meta-analysis protocols (PRISMA-P) 2015: elaboration and explanation. BMJ. 2015 Jan 2;349(jan02 1):g7647

For peer review only

BMJ Open

Machine learning health-related applications in low- and middle-income countries: A scoping review protocol

Journal:	*BMJ Open*
Manuscript ID	bmjopen-2019-035983.R2
Article Type:	Protocol
Date Submitted by the Author:	16-Apr-2020
Complete List of Authors:	Carrillo-Larco, Rodrigo; Universidad Peruana Cayetano Heredia, CRONICAS Center of Excellence; CRONICAS Center of Excellence in Chronic Diseases<br>Tudor Car, Lorainne; Lee Kong Chian School of Medicine, Family Medicine and Primary Care<br>Pearson-Stuttard, Jonathan; Imperial College London, PCPH<br>Panch, Trishan; Wellframe Inc.<br>Miranda, J. Jaime; Universidad Peruana Cayetano Heredia, CRONICAS Centre of Excellence in Chronic Diseases<br>Atun, Rifat; Harvard University, Harvard School of Public Health
**Primary Subject Heading**:	Global health
Secondary Subject Heading:	Diagnostics, Epidemiology
Keywords:	EPIDEMIOLOGY, BIOTECHNOLOGY & BIOINFORMATICS, Health informatics < BIOTECHNOLOGY & BIOINFORMATICS, World Wide Web technology < BIOTECHNOLOGY & BIOINFORMATICS

SCHOLARONE™
Manuscripts

I, the Submitting Author has the right to grant and does grant on behalf of all authors of the Work (as defined in the below author licence), an exclusive licence and/or a non-exclusive licence for contributions from authors who are: i) UK Crown employees; ii) where BMJ has agreed a CC-BY licence shall apply, and/or iii) in accordance with the terms applicable for US Federal Government officers or employees acting as part of their official duties; on a worldwide, perpetual, irrevocable, royalty-free basis to BMJ Publishing Group Ltd ("BMJ") its licensees and where the relevant Journal is co-owned by BMJ to the co-owners of the Journal, to publish the Work in this journal and any other BMJ products and to exploit all rights, as set out in our licence.

The Submitting Author accepts and understands that any supply made under these terms is made by BMJ to the Submitting Author unless you are acting as an employee on behalf of your employer or a postgraduate student of an affiliated institution which is paying any applicable article publishing charge ("APC") for Open Access articles. Where the Submitting Author wishes to make the Work available on an Open Access basis (and intends to pay the relevant APC), the terms of reuse of such Open Access shall be governed by a Creative Commons licence – details of these licences and which Creative Commons licence will apply to this Work are set out in our licence referred to above.

Other than as permitted in any relevant BMJ Author's Self Archiving Policies, I confirm this Work has not been accepted for publication elsewhere, is not being considered for publication elsewhere and does not duplicate material already published. I confirm all authors consent to publication of this Work and authorise the granting of this licence.

For peer review only

Machine learning health-related applications in low- and middle-income countries: A scoping review protocol

Rodrigo M Carrillo-Larco (r.carrillo-larco@imperial.ac.uk) [1,2]

Lorainne Tudor Car (lorainne.tudor.car@ntu.edu.sg) [3,4]

Jonathan Pearson-Stuttard (j.pearson-stuttard@imperial.ac.uk) [5]

Trishan Panch (trishan@wellframe.com) [6]

J Jaime Miranda (jaime.miranda@upch.pe) [2,7]

Rifat Atun (ratun@hsph.harvard.edu) [8]

1. Department of Epidemiology and Biostatistics, School of Public Health, Imperial College London, London, UK.
2. CRONICAS Centre of Excellence in Chronic Diseases, Universidad Peruana Cayetano Heredia, Lima, Peru.
3. Family Medicine and Primary Care, Lee Kong Chian School of Medicine, Nanyang Technological University, Singapore.
4. Department of Primary Care and Public Health, School of Public Health, Imperial College London, London, UK.
5. Department of Epidemiology and Biostatistics and MRC-PHE Centre for Environment and Health, School of Public Health, Imperial College London, London, UK.
6. Wellframe Inc., Boston, Massachusetts, USA.
7. Facultad de Medicina "Alberto Hurtado", Universidad Peruana Cayetano Heredia, Lima, Peru.
8. Harvard T.H Chan, School of Public Health, and Harvard Medical School, Harvard University, Cambridge, Massachusetts, USA.

For peer review only

For peer review only - http://bmjopen.bmj.com/site/about/guidelines.xhtml

Corresponding author:

Rodrigo M. Carrillo-Larco, MD

Department of Epidemiology and Biostatistics

School of Public Health

Imperial College London, London, UK

r.carrillo-larco@imperial.ac.uk

Key words: Artificial intelligence; Algorithms; Developing countries

Word count: 1,540

For peer review only

For peer review only - http://bmjopen.bmj.com/site/about/guidelines.xhtml

ABSTRACT

Introduction: Machine learning (ML) has been used in bio-medical research, and recently in clinical and public health research. However, much of the available evidence comes from high-income countries where different health profiles challenges the application of this research to low- and middle-income countries (LMICs). It is largely unknown what ML applications are available for LMICs that can support and advance clinical medicine and public health. We aim to address this gap by conducting a scoping review of health-related ML applications in LMICs.

Methods and analysis: This scoping review will follow the methodology proposed by Levac *et al*. The search strategy is informed by recent systematic reviews of ML health-related applications. We will search Embase, Medline and Global Health (through OVID), Cochrane and Google Scholar; we will present the date of our searches in the final review. Titles and abstracts will be screened by two reviewers independently; selected reports will be studied by two reviewers independently. Reports will be included if they are primary research where data have been analysed, ML techniques have been used on data from LMICs, and they aimed to improve health-related outcomes. We will synthesize the information following evidence mapping recommendations.

Ethics and dissemination: The review will provide a comprehensive list of health-related ML applications in LMICs. The results will be disseminated through scientific publications. We also plan to launch a website where ML models can be hosted so that researchers, policymakers and the general public can readily access them.

For peer review only

STRENGTHS AND LIMITATIONS OF THIS STUDY

- The search strategy is informed by a solid framework.
- The search will be conducted in several information sources.
- The screening/review process will be performed by two reviewers independently.
- We will not search abstracts or conference proceedings specialized in machine learning.
- We will focus on information sources where health-related outcomes are most likely to be found.

For peer review only

For peer review only - http://bmjopen.bmj.com/site/about/guidelines.xhtml

INTRODUCTION

Machine learning refers to the process through which computers, models or algorithms, learn and improve from data and processes, rather than from specific programmed instructions.[1,2] Machine learning can be used in tasks such as classification (e.g., whether a tumour is benign or malign based on patterns or characteristics), clustering (e.g., group patients with similar profiles for targeted prevention or treatment interventions) or prediction (e.g., forecast propensity to risk or probability of outcome of a disease following interventions).[2] Of note, there could be overlap across different tasks.

Machine learning is widely used in bio-medical sciences, and more recently in clinical and public health research.[3,4] Systematic reviews on health-related applications of machine learning have explored questions such as the accuracy of machine learning for diagnosis or outcome prediction,[5-7] but most of the research studies included in these reviews have come from high-income countries and the findings may not apply to low-income and middle-income countries (LMICs) because the variability in access to healthcare and difference in the disease burden. It is largely unknown what machine learning applications are available for LMICs that can support and advance clinical medicine and public health.

Our aim was to address this gap in the evidence by conducting a scoping review of health-related applications of machine learning in LMICs to synthesise published evidence and to garner lessons to inform further research and policy development. The review will provide the first comprehensive list of health-related machine learning applications in LMICs.

METHODS

Overview

This is a scoping review of the published scientific literature. This protocol adheres to the PRISMA-P guidelines (supplementary material S1),[8] and the methodology follows the procedures suggested by Levac et al.[9] The final publication of this work will adhere to the PRISMA-ScR recommendations.[10]

For peer review only - http://bmjopen.bmj.com/site/about/guidelines.xhtml

PRISMA-P is a standard method to report review protocols; similarly, PRSIMA-ScR is a well-known instrument to guide the reporting of scoping reviews. The methodology proposed by Levac and colleagues is useful for scoping reviews that aim to generate a broad picture of the available evidence on a subject.

Understanding that a key feature of scoping reviews is a broad research question, we aim to answer: *what have been the health-related applications of machine learning techniques in LMICs?* This review will summarize scientific evidence on research that have used data from LMICs for machine learning applications in clinical medicine (e.g., risk prediction for clinical decisions) and public health (e.g., vector control in an endemic area) to provide solutions to health problems in LMICs.

Definitions

For this work we will follow these definitions:

- LMICs: classification by the World Bank country income grouping (see search strategy in Table 1 for a list of countries).[11]
- Machine learning: analytical techniques through which computers learn directly from data, examples and experiences, rather than from a pre-programmed rule.[1,2]
- Machine learning techniques: analytical methods within the machine learning remit, i.e. where machines have learnt from data or processes through machine learning techniques or algorithms. These include, but are not limited to:[2,12] support vector machine, support vector regression, decision trees, random forest, neural network, Bayesian network, artificial neural network, computer vision systems, computer-assisted image processing and natural language processing.

Eligibility criteria

We will include scientific evidence that meets the following inclusion criteria (Figure 1):

For peer review only - http://bmjopen.bmj.com/site/about/guidelines.xhtml

Primary research studies.

We will include experimental and observational studies that have used and reported quantitative data. We will also screen relevant systematic reviews or narrative reviews for eligible primary studies and include them in our review if relevant. We will not include qualitative studies, opinions, conference abstracts, letters, editorials or any other scientific work in which data have not been actively analysed within a machine learning framework.

Machine learning research should have used data from LMICs, i.e. machine learning research that used solely data from LMICs.

We will focus on machine learning research based on data from LMICs applied to LMICs. We will not include machine learning research that used data from high-income countries even though it could have been applied to a LMIC, and neither studies using LMICs data applied to high-income countries. This scoping review focuses on the applications of machine learning techniques in LMICs; these applications should have been developed using LMICs data, because prediction models and other algorithms work better, i.e. have better accuracy, in the populations – data – for which they were developed. Conversely, when these models are applied to other populations, settings or data, they need some modification (e.g., re-calibration). We will also exclude studies that have used LMIC data in a consortium or data pooling group if the model or results cannot be separated for the LMIC alone; in other words, we will exclude a report if this used LMIC data in aggregate with data from high-income countries, but the application cannot be separated for the LMIC alone.

Models developed in sites other than LMICs may not work correctly in these countries. For example, projects with digital imaging from high-income countries may reflect a different scenario; i.e., images from streets in LMICs may depict objects or features not found in high-income countries. Another example could be projects for sound/noise classification. Those from high-income countries may not identify the variety of noises usually available in LIMCs (e.g., loud cars or indiscriminate use of car

For peer review only - http://bmjopen.bmj.com/site/about/guidelines.xhtml

horns). Finally, LMICs still have sizable rural areas with large populations. Extrapolating models built for highly urbanized cities may not be adequate for rural sites.

Examples of studies of interest include: i) development of a 'deep learning-based visual evaluation algorithm' to early identify cervical cancer signs based on data from women in Costa Rica.[13] ii) Classification of free-text (random forest) in emergency department records from nine hospitals in Nicaragua.[14] iii) Automatic classification (neural networks) of paediatric pneumonia based on ultrasound records from children in Peru.[15] During the selection stages.

Distinguishing between machine learning applications and more conventional statistical methods could be challenging because in some cases the definitions are unclear; for instance, regression analysis. Nonetheless, from the context of the scientific paper, from the aims or overall methodological approach, it is possible to reckon whether a study uses machine learning techniques versus more conventional statistical methods. If needed, we would reach out to authors for further information.

The outcome of the study/analysis was to improve a health-related outcome.

The *primary outcome* of the selected studies should have sought improvements in the following health-related endpoints along the care cascade: diagnosis, treatment, control, survival, complications and mortality. These outcomes have been selected because of their relevance in healthcare, clinical medicine and public health. As *secondary outcome* we will also include studies that have reported endpoints related to cost, efficiency and productivity in the healthcare process.

Information sources

We will conduct the search in three databases through Ovid: Embase, Medline and Global Health; in addition, we will search Cochrane and Google Scholar (first 10 pages). Besides Google Scholar, no other grey literature source will be searched. These sources will be used without language or time/year restrictions. We have a very diverse team covering many languages and we will tap into our

For peer review only - http://bmjopen.bmj.com/site/about/guidelines.xhtml

For peer review only

networks at our institutions in case we come across a study in a language that the authors do not speak.

Search strategy

Based on recent systematic reviews of machine learning applications on health-related endpoints: outcome prediction in gastrointestinal bleeding,[7] assessment of physicians knowledge,[16] applications on genomic data to predict outcomes in cancer patients,[17] and available machine learning algorithms to improve genomic data analysis,[18] we developed our search strategy (Table 1). We will screen the references of the included reports for any other relevant studies.

Study records

Results will be downloaded into EndNote where duplicates will be omitted, and a second cleaning of duplicated results will be conducted using the online tool Rayyan.[19] Titles and abstracts will be reviewed by two independent researchers following the above detailed selection criteria (Figure 1); discrepancies will be solved by consensus or by a third party. Selected titles will be sought in full-text, which will be assessed by two independent reviewers following the same selection criteria (Figure 1). Again, in case of conflicts, these will be solved by consensus or by a third party.

Data collection

The reviewers will decide on a list of items that will be extracted to successfully answer the research question. These items will be implemented in an Excel spreadsheet before data extraction and will not be modified afterwards. Information of interest includes: country of origin; analytical approach; type of data used; data source; model performance; outcome of interest; number of observations; and whether the model is available for independent use or reproduction. Data from the selected reports will be extracted by two researchers independently; if there were discrepancies, these will be solved by consensus among them or by a third party.

For peer review only

For peer review only - http://bmjopen.bmj.com/site/about/guidelines.xhtml

Risk of bias in individual studies

Because this is a scoping review aiming to summarise available evidence to identify research gaps and potential uses of machine learning to improve health-related outcomes in LMICs, no risk of bias of individual studies is planned.

Data synthesis

We anticipate a large heterogeneity of selected reports, both in terms of methodology and outcomes, as well as target population and data sources. Therefore, no meta-analysis is planned and only a qualitative summary will be conducted. Following current recommendations for scoping reviews and evidence mapping,[20] we will present the results through tables and figures, e.g. a map pointing out where studies have been conducted and summarizing key characteristics. As needed, we will consider other figures such as a matrix evidence.[20]

Patient and public involvement - Dissemination

We plan to report our findings in a scientific publication. In addition, and depending on available resources, we aim to produce a website (or implement in an existing website) in which the findings and summarized reports can be easily accessed. Furthermore, we aim to host the machine learning models so that researchers, policymakers and the general public can readily access them. Where the machine learning models are open access or can be accessed through the original reports, these will be hosted on the website or a link to the original source will be provided; conversely, where machine learning models are not open access, we will contact the study authors and ask for the model to be hosted in our website or for a link to their machine learning model. This dissemination plan aims to increase visibility of machine learning research in LMICs and to increase the use of available models, thereby encouraging further research to improve health outcomes. We will engage with the communication office in our universities to promote this website through relevant channels including but not limited to social media, newsletters and institutional websites.

For peer review only - http://bmjopen.bmj.com/site/about/guidelines.xhtml

CONTRIBUTION STATEMENT

RMC-L conceived the idea and drafted the manuscript. JP-S, TP and RA provided advice to improve the research question and LTC to improve the protocol. JJM and RA edited and provided insights to improve the protocol. All authors approved the submitted version.

COMPETING INTERESTS

None to declare.

FUNDING

RMC-L has been supported by a Strategic Award, Wellcome Trust-Imperial College Centre for Global Health Research (100693/Z/12/Z), and Imperial College London Wellcome Trust Institutional Strategic Support Fund [Global Health Clinical Research Training Fellowship] (294834/Z/16/Z ISSF ICL). RMC-L is supported by a Wellcome Trust International Training Fellowship (214185/Z/18/Z). The funders had no role in this work and decision to submit for publication.

DATA SHARING STATEMENT

Not applicable

For peer review only

For peer review only - http://bmjopen.bmj.com/site/about/guidelines.xhtml

REFERENCES

1. Davies, S.C. "Annual Report of the Chief Medical Officer, 2018 Health 2040 – Better Health Within Reach": Department of Health and Social Care (2018).

2. Rebala G, Ravi A, Churiwala S. An Introduction to Machine Learning: Springer International Publishing; 2019.

3. Panch T, Pearson-Stuttard J, Greaves F, Atun R. Artificial intelligence: opportunities and risks for public health. *The Lancet Digital Health* 2019; **1**(1): e13-e4.

4. Wahl B, Cossy-Gantner A, Germann S, Schwalbe NR. Artificial intelligence (AI) and global health: how can AI contribute to health in resource-poor settings? *BMJ global health* 2018; **3**(4): e000798.

5. de Filippis R, Carbone EA, Gaetano R, et al. Machine learning techniques in a structural and functional MRI diagnostic approach in schizophrenia: a systematic review. *Neuropsychiatric disease and treatment* 2019; **15**: 1605-27.

6. Bradley A, van der Meer R, McKay C. Personalized Pancreatic Cancer Management: A Systematic Review of How Machine Learning Is Supporting Decision-making. *Pancreas* 2019; **48**(5): 598-604.

7. Shung D, Simonov M, Gentry M, Au B, Laine L. Machine Learning to Predict Outcomes in Patients with Acute Gastrointestinal Bleeding: A Systematic Review. *Digestive diseases and sciences* 2019; **64**(8): 2078-87.

8. Shamseer L, Moher D, Clarke M, et al. Preferred reporting items for systematic review and meta-analysis protocols (PRISMA-P) 2015: elaboration and explanation. *BMJ (Clinical research ed)* 2015; **350**: g7647.

9. Levac D, Colquhoun H, O'Brien KK. Scoping studies: advancing the methodology. *Implementation science : IS* 2010; **5**: 69.

10. Tricco AC, Lillie E, Zarin W, et al. PRISMA Extension for Scoping Reviews (PRISMA-ScR): Checklist and Explanation. *Annals of internal medicine* 2018; **169**(7): 467-73.

For peer review only - http://bmjopen.bmj.com/site/about/guidelines.xhtml

11. The World Bank. World Bank Country and Lending Groups. URL: https://datahelpdesk.worldbank.org/knowledgebase/articles/906519-world-bank-country-and-lending-groups.

12. Burkov A. The Hundred-page Machine Learning Book: Andriy Burkov; 2019.

13. Hu L, Bell D, Antani S, et al. An Observational Study of Deep Learning and Automated Evaluation of Cervical Images for Cancer Screening. *Journal of the National Cancer Institute* 2019.

14. Lorenzoni G, Bressan S, Lanera C, Azzolina D, Da Dalt L, Gregori D. Analysis of Unstructured Text-Based Data Using Machine Learning Techniques: The Case of Pediatric Emergency Department Records in Nicaragua. *Medical care research and review : MCRR* 2019: 1077558719844123.

15. Correa M, Zimic M, Barrientos F, et al. Automatic classification of pediatric pneumonia based on lung ultrasound pattern recognition. *PloS one* 2018; **13**(12): e0206410.

16. Dias RD, Gupta A, Yule SJ. Using Machine Learning to Assess Physician Competence: A Systematic Review. *Academic medicine : journal of the Association of American Medical Colleges* 2019; **94**(3): 427-39.

17. Patil S, Habib Awan K, Arakeri G, et al. Machine learning and its potential applications to the genomic study of head and neck cancer-A systematic review. *Journal of oral pathology & medicine : official publication of the International Association of Oral Pathologists and the American Academy of Oral Pathology* 2019.

18. Wu J, Zhao Y. Machine learning technology in the application of genome analysis: A systematic review. *Gene* 2019; **705**: 149-56.

19. Ouzzani M, Hammady H, Fedorowicz Z, Elmagarmid A. Rayyan-a web and mobile app for systematic reviews. *Systematic reviews* 2016; **5**(1): 210.

20. Evidence and gap maps: a comparison of different approaches. Oslo, Norway: The Campbell Collaboration. Retrieved from: www.campbellcollaboration.org/ DOI: https://doi.org/10.4073/cmdp.2018.2.

For peer review only - http://bmjopen.bmj.com/site/about/guidelines.xhtml

TABLES

Table 1: Overall search terms

1	artificial intelligence.mp.
2	exp Artificial Intelligence/
3	machine learning.mp.
4	exp machine learning/
5	deep learning.mp.
6	unsupervised machine learning.mp.
7	supervised machine learning.mp.
8	computational Intelligence.mp.
9	predictive analytic*.mp.
10	support vector machine.mp.
11	support vector regression.mp.
12	decision tree*.mp.
13	random forest.mp.
14	neural network*.mp.
15	exp Neural Networks/
16	bayesian network*.mp.
17	artificial neural network*.mp.
18	convolutional neural network*.mp.
19	computer vision systems.mp.
20	exp Image Processing, Computer-Assisted/
21	natural language processesing.mp.
22	1 or 2 or 3 …or 21
23	(("Afghanistan") or ("Benin") or ("Burkina Faso") or ("Burundi") or ("Central African Republic") or ("Chad") or ("Comoros") or ("Democratic Republic of the Congo") or ("Eritrea") or ("Ethiopia") or ("Gambia") or ("Guinea") or ("Guinea-Bissau") or ("Haiti") or ("Democratic People's Republic of Korea") or ("Liberia") or ("Madagascar") or ("Malawi") or ("Mali") or ("Mozambique") or ("Nepal") or ("Niger") or ("Rwanda") or ("Senegal") or ("Sierra Leone") or ("Somalia") or ("South Sudan") or ("Tanzania") or ("Togo") or ("Uganda") or ("Zimbabwe") or ("Armenia") or ("Bangladesh") or ("Bhutan") or ("Bolivia") or ("Cape Verde") or ("Cambodia") or ("Cameroon") or ("Congo") or ("Cote d'Ivoire") or ("Djibouti") or ("Egypt") or ("El Salvador") or ("Ghana") or ("Guatemala") or ("Honduras") or ("India") or ("Indonesia") or ("Kenya") or ("Micronesia") or ("Kosovo") or ("Kyrgyzstan") or ("Laos") or ("Lesotho") or ("Mauritania") or ("Moldova") or ("Mongolia") or ("Morocco") or ("Myanmar") or ("Nicaragua") or ("Nigeria") or ("Pakistan") or ("Papua New Guinea") or ("Philippines") or ("Samoa") or ("Atlantic Islands") or ("Melanesia") or ("Sri Lanka") or ("Sudan") or ("Swaziland") or ("Syria") or ("Tajikistan") or ("Timor-Leste") or ("Tonga") or ("Tunisia") or ("Ukraine") or ("Uzbekistan") or ("Vanuatu") or ("Vietnam") or ("Middle East") or ("Yemen") or ("Zambia") or ("Albania") or ("Algeria") or ("American Samoa") or ("Angola") or ("Argentina") or ("Azerbaijan") or ("Republic of Belarus") or ("Belize") or ("Bosnia and Herzegovina") or ("Botswana") or ("Brazil") or ("Bulgaria") or ("China") or ("Colombia") or ("Costa Rica") or ("Cuba") or ("Dominica") or ("Dominican Republic") or ("Equatorial Guinea") or ("Ecuador") or ("Fiji") or ("Gabon") or ("Georgia") or ("Grenada") or ("Guyana") or ("Iran") or ("Iraq") or ("Jamaica") or ("Jordan") or ("Kazakhstan") or ("Lebanon") or ("Libya") or ("Macedonia (Republic)") or ("Malaysia") or ("Indian Ocean Islands") or ("Mauritius") or ("Mexico") or ("Montenegro") or ("Namibia") or ("Palau") or

For peer review only

For peer review only - http://bmjopen.bmj.com/site/about/guidelines.xhtml

	("Panama") or ("Paraguay") or ("Peru") or ("Romania") or ("Russia") or ("Serbia") or ("South Africa") or ("Saint Lucia") or ("Saint Vincent and the Grenadines") or ("Suriname") or ("Thailand") or ("Turkey") or ("Turkmenistan") or ("Venezuela") or (developing countr) or (low-income countr*) or (middle-income countr*) or (low-middle income countr*) or (upper-middle income countr*))
24	22 and 23
25	exp animals/ not humans.sh.
26	24 not 25
27	Remove duplicates from 26

For peer review only

For peer review only - http://bmjopen.bmj.com/site/about/guidelines.xhtml

FIGURES

Figure 1: Algorithm to filter search results

For peer review only

For peer review only - http://bmjopen.bmj.com/site/about/guidelines.xhtml

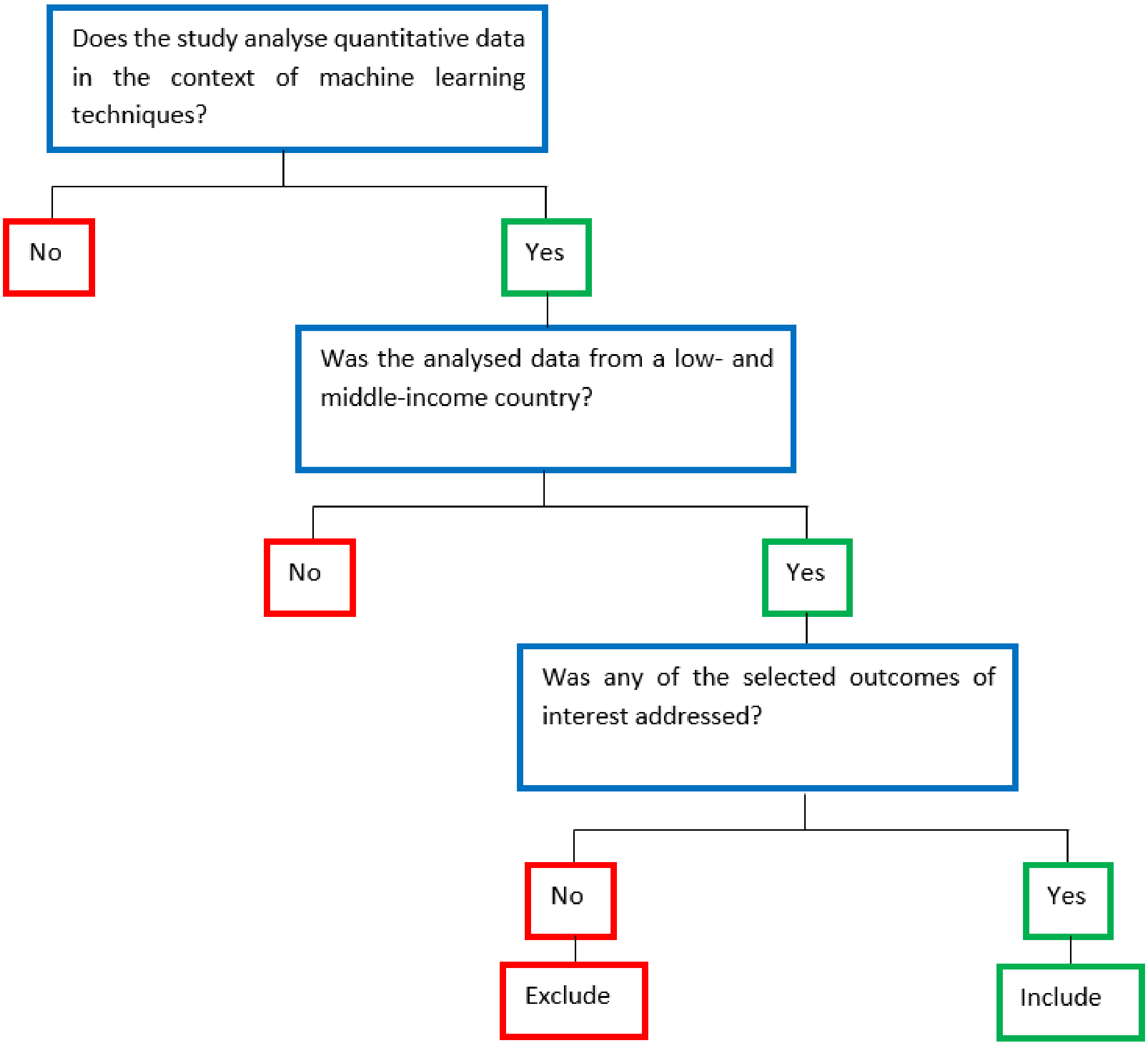

For peer review only - http://bmjopen.bmj.com/site/about/guidelines.xhtml

SUPPLEMENTARY MATERIAL

A. *S1: PRISMA-P checklist*

PRISMA-P (Preferred Reporting Items for Systematic review and Meta-Analysis Protocols) 2015 checklist: recommended items to address in a systematic review protocol

Section and topic	Item No	Checklist item	Page	Paragraph
**ADMINISTRATIVE INFORMATION**				
Title:				
Identification	1a	Identify the report as a protocol of a systematic review	1	1
Update	1b	If the protocol is for an update of a previous systematic review, identify as such	NA	NA
Registration	2	If registered, provide the name of the registry (such as PROSPERO) and registration number	NA	NA
Authors:				
Contact	3a	Provide name, institutional affiliation, e-mail address of all protocol authors; provide physical mailing address of corresponding author	1	1
Contributions	3b	Describe contributions of protocol authors and identify the guarantor of the review	2	3
Amendments	4	If the protocol represents an amendment of a previously completed or published protocol, identify as such and list changes; otherwise, state plan for documenting important protocol amendments	NA	NA
Support:				
Sources	5a	Indicate sources of financial or other support for the review	2	2
Sponsor	5b	Provide name for the review funder and/or sponsor	2	2
Role of sponsor or funder	5c	Describe roles of funder(s), sponsor(s), and/or institution(s), if any, in developing the protocol	2	2
**INTRODUCTION**				
Rationale	6	Describe the rationale for the review in the context of what is already known	5	2

For peer review only

Objectives	7	Provide an explicit statement of the question(s) the review will address with reference to participants, interventions, comparators, and outcomes (PICO)	5	3
**METHODS**				
Eligibility criteria	8	Specify the study characteristics (such as PICO, study design, setting, time frame) and report characteristics (such as years considered, language, publication status) to be used as criteria for eligibility for the review	6	6
Information sources	9	Describe all intended information sources (such as electronic databases, contact with study authors, trial registers or other grey literature sources) with planned dates of coverage	8	2
Search strategy	10	Present draft of search strategy to be used for at least one electronic database, including planned limits, such that it could be repeated	8	3
Study records:				
Data management	11a	Describe the mechanism(s) that will be used to manage records and data throughout the review	8	4
Selection process	11b	State the process that will be used for selecting studies (such as two independent reviewers) through each phase of the review (that is, screening, eligibility and inclusion in meta-analysis)	8	5
Data collection process	11c	Describe planned method of extracting data from reports (such as piloting forms, done independently, in duplicate), any processes for obtaining and confirming data from investigators	8	5
Data items	12	List and define all variables for which data will be sought (such as PICO items, funding sources), any pre-planned data assumptions and simplifications	NA	NA
Outcomes and prioritization	13	List and define all outcomes for which data will be sought, including prioritization of main and additional outcomes, with rationale	7	4
Risk of bias in individual studies	14	Describe anticipated methods for assessing risk of bias of individual studies, including whether this will be done at the outcome or study level, or both; state how this information will be used in data synthesis	9	2
Data synthesis	15a	Describe criteria under which study data will be quantitatively synthesised	9	3
	15b	If data are appropriate for quantitative synthesis, describe planned summary measures, methods of handling data and methods of combining data from studies, including any planned exploration of consistency (such as I^2, Kendall's τ)	NA	NA
	15c	Describe any proposed additional analyses (such as sensitivity or subgroup analyses, meta-regression)	NA	NA
	15d	If quantitative synthesis is not appropriate, describe the type of summary planned	NA	NA

For peer review only

Meta-bias(es)	16	Specify any planned assessment of meta-bias(es) (such as publication bias across studies, selective reporting within studies)	NA	NA
Confidence in cumulative evidence	17	Describe how the strength of the body of evidence will be assessed (such as GRADE)	NA	NA

From: Shamseer L, Moher D, Clarke M, Ghersi D, Liberati A, Petticrew M, Shekelle P, Stewart L, PRISMA-P Group. Preferred reporting items for systematic review and meta-analysis protocols (PRISMA-P) 2015: elaboration and explanation. BMJ. 2015 Jan 2;349(jan02 1):g7647

For peer review only

BMJ Open

Machine learning health-related applications in low- and middle-income countries: A scoping review protocol

Journal:	*BMJ Open*
Manuscript ID	bmjopen-2019-035983.R3
Article Type:	Protocol
Date Submitted by the Author:	17-Apr-2020
Complete List of Authors:	Carrillo-Larco, Rodrigo; Universidad Peruana Cayetano Heredia, CRONICAS Center of Excellence; CRONICAS Center of Excellence in Chronic Diseases<br>Tudor Car, Lorainne; Lee Kong Chian School of Medicine, Family Medicine and Primary Care<br>Pearson-Stuttard, Jonathan; Imperial College London, PCPH<br>Panch, Trishan; Wellframe Inc.<br>Miranda, J. Jaime; Universidad Peruana Cayetano Heredia, CRONICAS Centre of Excellence in Chronic Diseases<br>Atun, Rifat; Harvard University, Harvard School of Public Health
**Primary Subject Heading**:	Global health
Secondary Subject Heading:	Diagnostics, Epidemiology
Keywords:	EPIDEMIOLOGY, BIOTECHNOLOGY & BIOINFORMATICS, Health informatics < BIOTECHNOLOGY & BIOINFORMATICS, World Wide Web technology < BIOTECHNOLOGY & BIOINFORMATICS

SCHOLARONE™
Manuscripts

I, the Submitting Author has the right to grant and does grant on behalf of all authors of the Work (as defined in the below author licence), an exclusive licence and/or a non-exclusive licence for contributions from authors who are: i) UK Crown employees; ii) where BMJ has agreed a CC-BY licence shall apply, and/or iii) in accordance with the terms applicable for US Federal Government officers or employees acting as part of their official duties; on a worldwide, perpetual, irrevocable, royalty-free basis to BMJ Publishing Group Ltd ("BMJ") its licensees and where the relevant Journal is co-owned by BMJ to the co-owners of the Journal, to publish the Work in this journal and any other BMJ products and to exploit all rights, as set out in our licence.

The Submitting Author accepts and understands that any supply made under these terms is made by BMJ to the Submitting Author unless you are acting as an employee on behalf of your employer or a postgraduate student of an affiliated institution which is paying any applicable article publishing charge ("APC") for Open Access articles. Where the Submitting Author wishes to make the Work available on an Open Access basis (and intends to pay the relevant APC), the terms of reuse of such Open Access shall be governed by a Creative Commons licence – details of these licences and which Creative Commons licence will apply to this Work are set out in our licence referred to above.

Other than as permitted in any relevant BMJ Author's Self Archiving Policies, I confirm this Work has not been accepted for publication elsewhere, is not being considered for publication elsewhere and does not duplicate material already published. I confirm all authors consent to publication of this Work and authorise the granting of this licence.

For peer review only

Machine learning health-related applications in low- and middle-income countries: A scoping review protocol

Rodrigo M Carrillo-Larco (r.carrillo-larco@imperial.ac.uk) [1,2]

Lorainne Tudor Car (lorainne.tudor.car@ntu.edu.sg) [3,4]

Jonathan Pearson-Stuttard (j.pearson-stuttard@imperial.ac.uk) [5]

Trishan Panch (trishan@wellframe.com) [6]

J Jaime Miranda (jaime.miranda@upch.pe) [2,7]

Rifat Atun (ratun@hsph.harvard.edu) [8]

1. Department of Epidemiology and Biostatistics, School of Public Health, Imperial College London, London, UK.
2. CRONICAS Centre of Excellence in Chronic Diseases, Universidad Peruana Cayetano Heredia, Lima, Peru.
3. Family Medicine and Primary Care, Lee Kong Chian School of Medicine, Nanyang Technological University, Singapore.
4. Department of Primary Care and Public Health, School of Public Health, Imperial College London, London, UK.
5. Department of Epidemiology and Biostatistics and MRC-PHE Centre for Environment and Health, School of Public Health, Imperial College London, London, UK.
6. Wellframe Inc., Boston, Massachusetts, USA.
7. Facultad de Medicina "Alberto Hurtado", Universidad Peruana Cayetano Heredia, Lima, Peru.
8. Harvard T.H Chan, School of Public Health, and Harvard Medical School, Harvard University, Cambridge, Massachusetts, USA.

For peer review only

For peer review only - http://bmjopen.bmj.com/site/about/guidelines.xhtml

Corresponding author:

Rodrigo M. Carrillo-Larco, MD

Department of Epidemiology and Biostatistics

School of Public Health

Imperial College London, London, UK

r.carrillo-larco@imperial.ac.uk

Key words: Artificial intelligence; Algorithms; Developing countries

Word count: 1,540

For peer review only

For peer review only - http://bmjopen.bmj.com/site/about/guidelines.xhtml

ABSTRACT

Introduction: Machine learning (ML) has been used in bio-medical research, and recently in clinical and public health research. However, much of the available evidence comes from high-income countries where different health profiles challenges the application of this research to low- and middle-income countries (LMICs). It is largely unknown what ML applications are available for LMICs that can support and advance clinical medicine and public health. We aim to address this gap by conducting a scoping review of health-related ML applications in LMICs.

Methods and analysis: This scoping review will follow the methodology proposed by Levac *et al*. The search strategy is informed by recent systematic reviews of ML health-related applications. We will search Embase, Medline and Global Health (through OVID), Cochrane and Google Scholar; we will present the date of our searches in the final review. Titles and abstracts will be screened by two reviewers independently; selected reports will be studied by two reviewers independently. Reports will be included if they are primary research where data have been analysed, ML techniques have been used on data from LMICs, and they aimed to improve health-related outcomes. We will synthesize the information following evidence mapping recommendations.

Ethics and dissemination: The review will provide a comprehensive list of health-related ML applications in LMICs. The results will be disseminated through scientific publications. We also plan to launch a website where ML models can be hosted so that researchers, policymakers and the general public can readily access them.

For peer review only

For peer review only - http://bmjopen.bmj.com/site/about/guidelines.xhtml

STRENGTHS AND LIMITATIONS OF THIS STUDY

- The search strategy is informed by a solid framework.
- The search will be conducted in several information sources.
- The screening/review process will be performed by two reviewers independently.
- We will not search abstracts or conference proceedings specialized in machine learning.
- We will focus on information sources where health-related outcomes are most likely to be found.

For peer review only

For peer review only - http://bmjopen.bmj.com/site/about/guidelines.xhtml

INTRODUCTION

Machine learning refers to the process through which computers, models or algorithms, learn and improve from data and processes, rather than from specific programmed instructions.[1,2] Machine learning can be used in tasks such as classification (e.g., whether a tumour is benign or malign based on patterns or characteristics), clustering (e.g., group patients with similar profiles for targeted prevention or treatment interventions) or prediction (e.g., forecast propensity to risk or probability of outcome of a disease following interventions).[2] Of note, there could be overlap across different tasks.

Machine learning is widely used in bio-medical sciences, and more recently in clinical and public health research.[3,4] Systematic reviews on health-related applications of machine learning have explored questions such as the accuracy of machine learning for diagnosis or outcome prediction,[5-7] but most of the research studies included in these reviews have come from high-income countries and the findings may not apply to low-income and middle-income countries (LMICs) because the variability in access to healthcare and difference in the disease burden. It is largely unknown what machine learning applications are available for LMICs that can support and advance clinical medicine and public health.

Our aim was to address this gap in the evidence by conducting a scoping review of health-related applications of machine learning in LMICs to synthesise published evidence and to garner lessons to inform further research and policy development. The review will provide the first comprehensive list of health-related machine learning applications in LMICs.

METHODS

Overview

This is a scoping review of the published scientific literature. This protocol adheres to the PRISMA-P guidelines (supplementary material S1),[8] and the methodology follows the procedures suggested by Levac et al.[9] The final publication of this work will adhere to the PRISMA-ScR recommendations.[10]

For peer review only - http://bmjopen.bmj.com/site/about/guidelines.xhtml

PRISMA-P is a standard method to report review protocols; similarly, PRSIMA-ScR is a well-known instrument to guide the reporting of scoping reviews. The methodology proposed by Levac and colleagues is useful for scoping reviews that aim to generate a broad picture of the available evidence on a subject.

Understanding that a key feature of scoping reviews is a broad research question, we aim to answer: *what have been the health-related applications of machine learning techniques in LMICs?* This review will summarize scientific evidence on research that have used data from LMICs for machine learning applications in clinical medicine (e.g., risk prediction for clinical decisions) and public health (e.g., vector control in an endemic area) to provide solutions to health problems in LMICs.

For peer review only

Definitions

For this work we will follow these definitions:

- LMICs: classification by the World Bank country income grouping (see search strategy in Table 1 for a list of countries).[11]
- Machine learning: analytical techniques through which computers learn directly from data, examples and experiences, rather than from a pre-programmed rule.[1,2]
- Machine learning techniques: analytical methods within the machine learning remit, i.e. where machines have learnt from data or processes through machine learning techniques or algorithms. These include, but are not limited to:[2,12] support vector machine, support vector regression, decision trees, random forest, neural network, Bayesian network, artificial neural network, computer vision systems, computer-assisted image processing and natural language processing.

Eligibility criteria

We will include scientific evidence that meets the following inclusion criteria (Figure 1):

For peer review only - http://bmjopen.bmj.com/site/about/guidelines.xhtml

Primary research studies.

We will include experimental and observational studies that have used and reported quantitative data. We will also screen relevant systematic reviews or narrative reviews for eligible primary studies and include them in our review if relevant. We will not include qualitative studies, opinions, conference abstracts, letters, editorials or any other scientific work in which data have not been actively analysed within a machine learning framework.

Machine learning research should have used data from LMICs, i.e. machine learning research that used solely data from LMICs.

We will focus on machine learning research based on data from LMICs applied to LMICs. We will not include machine learning research that used data from high-income countries even though it could have been applied to a LMIC, and neither studies using LMICs data applied to high-income countries. This scoping review focuses on the applications of machine learning techniques in LMICs; these applications should have been developed using LMICs data, because prediction models and other algorithms work better, i.e. have better accuracy, in the populations – data – for which they were developed. Conversely, when these models are applied to other populations, settings or data, they need some modification (e.g., re-calibration). We will also exclude studies that have used LMIC data in a consortium or data pooling group if the model or results cannot be separated for the LMIC alone; in other words, we will exclude a report if this used LMIC data in aggregate with data from high-income countries, but the application cannot be separated for the LMIC alone.

Models developed in sites other than LMICs may not work correctly in these countries. For example, projects with digital imaging from high-income countries may reflect a different scenario; i.e., images from streets in LMICs may depict objects or features not found in high-income countries. Another example could be projects for sound/noise classification. Those from high-income countries may not identify the variety of noises usually available in LIMCs (e.g., loud cars or indiscriminate use of car

For peer review only - http://bmjopen.bmj.com/site/about/guidelines.xhtml

horns). Finally, LMICs still have sizable rural areas with large populations. Extrapolating models built for highly urbanized cities may not be adequate for rural sites.

Examples of studies of interest include: i) development of a 'deep learning-based visual evaluation algorithm' to early identify cervical cancer signs based on data from women in Costa Rica.[13] ii) Classification of free-text (random forest) in emergency department records from nine hospitals in Nicaragua.[14] iii) Automatic classification (neural networks) of paediatric pneumonia based on ultrasound records from children in Peru.[15] During the selection stages.

Distinguishing between machine learning applications and more conventional statistical methods could be challenging because in some cases the definitions are unclear; for instance, regression analysis. Nonetheless, from the context of the scientific paper, from the aims or overall methodological approach, it is possible to reckon whether a study uses machine learning techniques versus more conventional statistical methods. If needed, we would reach out to authors for further information.

The outcome of the study/analysis was to improve a health-related outcome.

The *primary outcome* of the selected studies should have sought improvements in the following health-related endpoints along the care cascade: diagnosis, treatment, control, survival, complications and mortality. These outcomes have been selected because of their relevance in healthcare, clinical medicine and public health. As *secondary outcome* we will also include studies that have reported endpoints related to cost, efficiency and productivity in the healthcare process.

Information sources

We will conduct the search in three databases through Ovid: Embase, Medline and Global Health; in addition, we will search Cochrane and Google Scholar (first 10 pages). Besides Google Scholar, no other grey literature source will be searched. These sources will be used without language or time/year restrictions. We have a very diverse team covering many languages and we will tap into

For peer review only - http://bmjopen.bmj.com/site/about/guidelines.xhtml

For peer review only

our networks at our institutions in case we come across a study in a language that the authors do not speak.

Search strategy

Based on recent systematic reviews of machine learning applications on health-related endpoints: outcome prediction in gastrointestinal bleeding,[7] assessment of physicians knowledge,[16] applications on genomic data to predict outcomes in cancer patients,[17] and available machine learning algorithms to improve genomic data analysis,[18] we developed our search strategy (Table 1). We will screen the references of the included reports for any other relevant studies.

Study records

Results will be downloaded into EndNote where duplicates will be omitted, and a second cleaning of duplicated results will be conducted using the online tool Rayyan.[19] Titles and abstracts will be reviewed by two independent researchers following the above detailed selection criteria (Figure 1); discrepancies will be solved by consensus or by a third party. Selected titles will be sought in full-text, which will be assessed by two independent reviewers following the same selection criteria (Figure 1). Again, in case of conflicts, these will be solved by consensus or by a third party.

Data collection

The reviewers will decide on a list of items that will be extracted to successfully answer the research question. These items will be implemented in an Excel spreadsheet before data extraction and will not be modified afterwards. Information of interest includes: country of origin; analytical approach; type of data used; data source; model performance; outcome of interest; number of observations; and whether the model is available for independent use or reproduction. Data from the selected reports will be extracted by two researchers independently; if there were discrepancies, these will be solved by consensus among them or by a third party.

For peer review only - http://bmjopen.bmj.com/site/about/guidelines.xhtml

For peer review only

Risk of bias in individual studies

Because this is a scoping review aiming to summarise available evidence to identify research gaps and potential uses of machine learning to improve health-related outcomes in LMICs, no risk of bias of individual studies is planned.

Data synthesis

We anticipate a large heterogeneity of selected reports, both in terms of methodology and outcomes, as well as target population and data sources. Therefore, no meta-analysis is planned and only a qualitative summary will be conducted. Following current recommendations for scoping reviews and evidence mapping,[20] we will present the results through tables and figures, e.g. a map pointing out where studies have been conducted and summarizing key characteristics. As needed, we will consider other figures such as a matrix evidence.[20]

Ethics and dissemination

This scoping review will not require ethical approval because it did not study human subjects; also, it included sources that are or can be made available to the public.

We plan to report our findings in a scientific publication. In addition, and depending on available resources, we aim to produce a website (or implement in an existing website) in which the findings and summarized reports can be easily accessed. Furthermore, we aim to host the machine learning models so that researchers, policymakers and the general public can readily access them. Where the machine learning models are open access or can be accessed through the original reports, these will be hosted on the website or a link to the original source will be provided; conversely, where machine learning models are not open access, we will contact the study authors and ask for the model to be hosted in our website or for a link to their machine learning model. This dissemination plan aims to increase visibility of machine learning research in LMICs and to increase the use of available models, thereby encouraging further research to improve health outcomes. We will engage with the

For peer review only - http://bmjopen.bmj.com/site/about/guidelines.xhtml

For peer review only

communication office in our universities to promote this website through relevant channels including but not limited to social media, newsletters and institutional websites.

Patient and public involvement

No patients will be directly involved in the design, planning and conception of this study.

For peer review only

CONTRIBUTION STATEMENT

RMC-L conceived the idea and drafted the manuscript. JP-S, TP and RA provided advice to improve the research question and LTC to improve the protocol. JJM and RA edited and provided insights to improve the protocol. All authors approved the submitted version.

COMPETING INTERESTS

None to declare.

FUNDING

RMC-L has been supported by a Strategic Award, Wellcome Trust-Imperial College Centre for Global Health Research (100693/Z/12/Z), and Imperial College London Wellcome Trust Institutional Strategic Support Fund [Global Health Clinical Research Training Fellowship] (294834/Z/16/Z ISSF ICL). RMC-L is supported by a Wellcome Trust International Training Fellowship (214185/Z/18/Z). The funders had no role in this work and decision to submit for publication.

DATA SHARING STATEMENT

Not applicable

For peer review only

REFERENCES

1. Davies, S.C. "Annual Report of the Chief Medical Officer, 2018 Health 2040 – Better Health Within Reach": Department of Health and Social Care (2018).

2. Rebala G, Ravi A, Churiwala S. An Introduction to Machine Learning: Springer International Publishing; 2019.

3. Panch T, Pearson-Stuttard J, Greaves F, Atun R. Artificial intelligence: opportunities and risks for public health. *The Lancet Digital Health* 2019; **1**(1): e13-e4.

4. Wahl B, Cossy-Gantner A, Germann S, Schwalbe NR. Artificial intelligence (AI) and global health: how can AI contribute to health in resource-poor settings? *BMJ global health* 2018; **3**(4): e000798.

5. de Filippis R, Carbone EA, Gaetano R, et al. Machine learning techniques in a structural and functional MRI diagnostic approach in schizophrenia: a systematic review. *Neuropsychiatric disease and treatment* 2019; **15**: 1605-27.

6. Bradley A, van der Meer R, McKay C. Personalized Pancreatic Cancer Management: A Systematic Review of How Machine Learning Is Supporting Decision-making. *Pancreas* 2019; **48**(5): 598-604.

7. Shung D, Simonov M, Gentry M, Au B, Laine L. Machine Learning to Predict Outcomes in Patients with Acute Gastrointestinal Bleeding: A Systematic Review. *Digestive diseases and sciences* 2019; **64**(8): 2078-87.

8. Shamseer L, Moher D, Clarke M, et al. Preferred reporting items for systematic review and meta-analysis protocols (PRISMA-P) 2015: elaboration and explanation. *BMJ (Clinical research ed)* 2015; **350**: g7647.

9. Levac D, Colquhoun H, O'Brien KK. Scoping studies: advancing the methodology. *Implementation science : IS* 2010; **5**: 69.

10. Tricco AC, Lillie E, Zarin W, et al. PRISMA Extension for Scoping Reviews (PRISMA-ScR): Checklist and Explanation. *Annals of internal medicine* 2018; **169**(7): 467-73.

For peer review only - http://bmjopen.bmj.com/site/about/guidelines.xhtml

11. The World Bank. World Bank Country and Lending Groups. URL: https://datahelpdesk.worldbank.org/knowledgebase/articles/906519-world-bank-country-and-lending-groups.

12. Burkov A. The Hundred-page Machine Learning Book: Andriy Burkov; 2019.

13. Hu L, Bell D, Antani S, et al. An Observational Study of Deep Learning and Automated Evaluation of Cervical Images for Cancer Screening. *Journal of the National Cancer Institute* 2019.

14. Lorenzoni G, Bressan S, Lanera C, Azzolina D, Da Dalt L, Gregori D. Analysis of Unstructured Text-Based Data Using Machine Learning Techniques: The Case of Pediatric Emergency Department Records in Nicaragua. *Medical care research and review : MCRR* 2019: 1077558719844123.

15. Correa M, Zimic M, Barrientos F, et al. Automatic classification of pediatric pneumonia based on lung ultrasound pattern recognition. *PloS one* 2018; **13**(12): e0206410.

16. Dias RD, Gupta A, Yule SJ. Using Machine Learning to Assess Physician Competence: A Systematic Review. *Academic medicine : journal of the Association of American Medical Colleges* 2019; **94**(3): 427-39.

17. Patil S, Habib Awan K, Arakeri G, et al. Machine learning and its potential applications to the genomic study of head and neck cancer-A systematic review. *Journal of oral pathology & medicine : official publication of the International Association of Oral Pathologists and the American Academy of Oral Pathology* 2019.

18. Wu J, Zhao Y. Machine learning technology in the application of genome analysis: A systematic review. *Gene* 2019; **705**: 149-56.

19. Ouzzani M, Hammady H, Fedorowicz Z, Elmagarmid A. Rayyan-a web and mobile app for systematic reviews. *Systematic reviews* 2016; **5**(1): 210.

20. Evidence and gap maps: a comparison of different approaches. Oslo, Norway: The Campbell Collaboration. Retrieved from: www.campbellcollaboration.org/ DOI: https://doi.org/10.4073/cmdp.2018.2.

For peer review only

For peer review only - http://bmjopen.bmj.com/site/about/guidelines.xhtml

TABLES

Table 1: Overall search terms

1	artificial intelligence.mp.
2	exp Artificial Intelligence/
3	machine learning.mp.
4	exp machine learning/
5	deep learning.mp.
6	unsupervised machine learning.mp.
7	supervised machine learning.mp.
8	computational Intelligence.mp.
9	predictive analytic*.mp.
10	support vector machine.mp.
11	support vector regression.mp.
12	decision tree*.mp.
13	random forest.mp.
14	neural network*.mp.
15	exp Neural Networks/
16	bayesian network*.mp.
17	artificial neural network*.mp.
18	convolutional neural network*.mp.
19	computer vision systems.mp.
20	exp Image Processing, Computer-Assisted/
21	natural language processesing.mp.
22	1 or 2 or 3 …or 21
23	(("Afghanistan") or ("Benin") or ("Burkina Faso") or ("Burundi") or ("Central African Republic") or ("Chad") or ("Comoros") or ("Democratic Republic of the Congo") or ("Eritrea") or ("Ethiopia") or ("Gambia") or ("Guinea") or ("Guinea-Bissau") or ("Haiti") or ("Democratic People's Republic of Korea") or ("Liberia") or ("Madagascar") or ("Malawi") or ("Mali") or ("Mozambique") or ("Nepal") or ("Niger") or ("Rwanda") or ("Senegal") or ("Sierra Leone") or ("Somalia") or ("South Sudan") or ("Tanzania") or ("Togo") or ("Uganda") or ("Zimbabwe") or ("Armenia") or ("Bangladesh") or ("Bhutan") or ("Bolivia") or ("Cape Verde") or ("Cambodia") or ("Cameroon") or ("Congo") or ("Cote d'Ivoire") or ("Djibouti") or ("Egypt") or ("El Salvador") or ("Ghana") or ("Guatemala") or ("Honduras") or ("India") or ("Indonesia") or ("Kenya") or ("Micronesia") or ("Kosovo") or ("Kyrgyzstan") or ("Laos") or ("Lesotho") or ("Mauritania") or ("Moldova") or ("Mongolia") or ("Morocco") or ("Myanmar") or ("Nicaragua") or ("Nigeria") or ("Pakistan") or ("Papua New Guinea") or ("Philippines") or ("Samoa") or ("Atlantic Islands") or ("Melanesia") or ("Sri Lanka") or ("Sudan") or ("Swaziland") or ("Syria") or ("Tajikistan") or ("Timor-Leste") or ("Tonga") or ("Tunisia") or ("Ukraine") or ("Uzbekistan") or ("Vanuatu") or ("Vietnam") or ("Middle East") or ("Yemen") or ("Zambia") or ("Albania") or ("Algeria") or ("American Samoa") or ("Angola") or ("Argentina") or ("Azerbaijan") or ("Republic of Belarus") or ("Belize") or ("Bosnia and Herzegovina") or ("Botswana") or ("Brazil") or ("Bulgaria") or ("China") or ("Colombia") or ("Costa Rica") or ("Cuba") or ("Dominica") or ("Dominican Republic") or ("Equatorial Guinea") or ("Ecuador") or ("Fiji") or ("Gabon") or ("Georgia") or ("Grenada") or ("Guyana") or ("Iran") or ("Iraq") or ("Jamaica") or ("Jordan") or ("Kazakhstan") or ("Lebanon") or ("Libya") or ("Macedonia (Republic)") or ("Malaysia") or ("Indian Ocean Islands") or ("Mauritius") or

For peer review only - http://bmjopen.bmj.com/site/about/guidelines.xhtml

	("Mexico") or ("Montenegro") or ("Namibia") or ("Palau") or ("Panama") or ("Paraguay") or ("Peru") or ("Romania") or ("Russia") or ("Serbia") or ("South Africa") or ("Saint Lucia") or ("Saint Vincent and the Grenadines") or ("Suriname") or ("Thailand") or ("Turkey") or ("Turkmenistan") or ("Venezuela") or (developing countr) or (low-income countr*) or (middle-income countr*) or (low-middle income countr*) or (upper-middle income countr*))
24	22 and 23
25	exp animals/ not humans.sh.
26	24 not 25
27	Remove duplicates from 26

For peer review only

For peer review only - http://bmjopen.bmj.com/site/about/guidelines.xhtml

FIGURES

Figure 1: Algorithm to filter search results

For peer review only

For peer review only - http://bmjopen.bmj.com/site/about/guidelines.xhtml

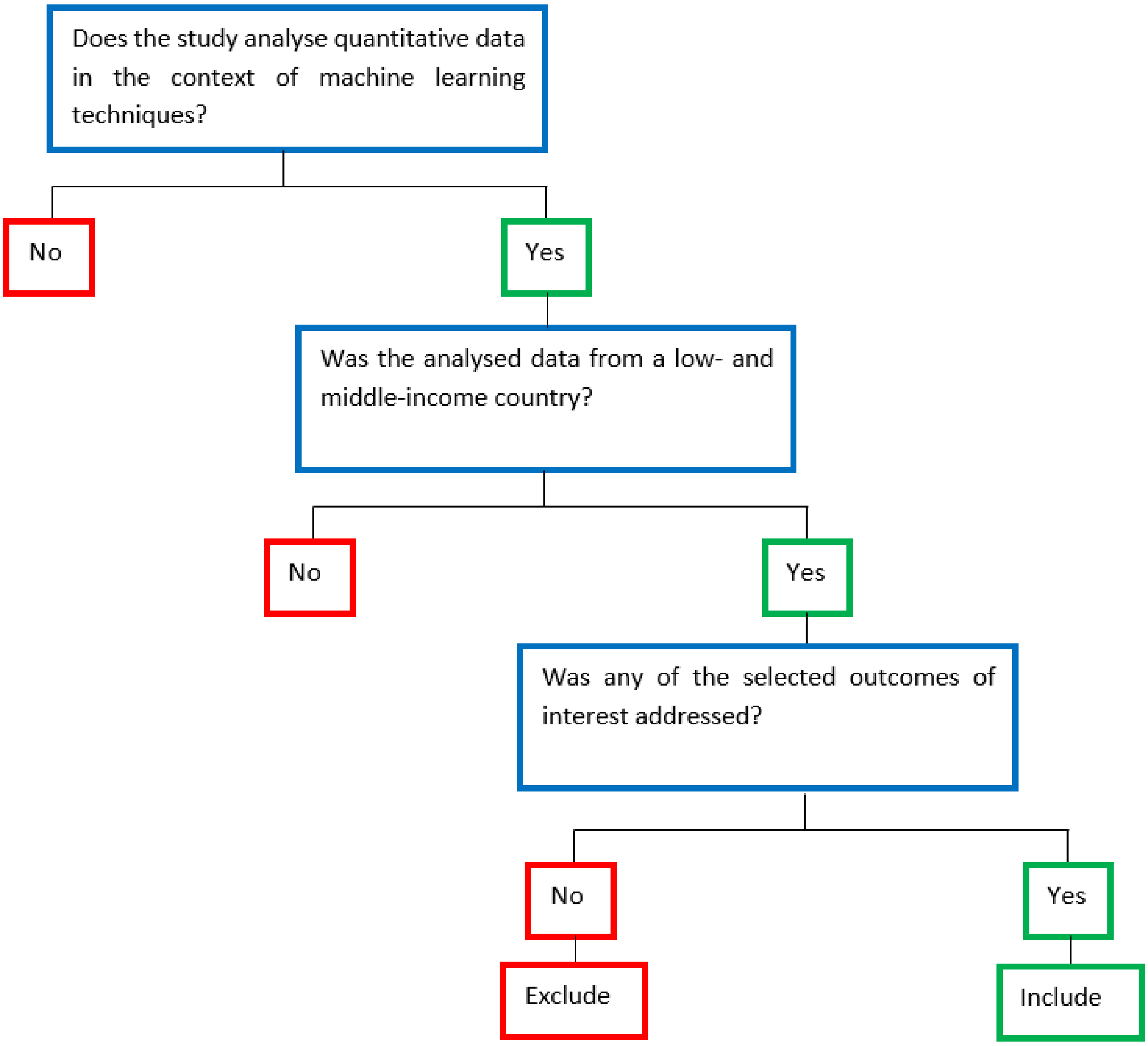
Does the study analyse quantitative data in the context of machine learning techniques?
No
Yes
Was the analysed data from a low- and middle-income country?
No
Yes
Was any of the selected outcomes of interest addressed?
No
Yes
Exclude
Include

SUPPLEMENTARY MATERIAL

A. *S1: PRISMA-P checklist*

PRISMA-P (Preferred Reporting Items for Systematic review and Meta-Analysis Protocols) 2015 checklist: recommended items to address in a systematic review protocol

Section and topic	Item No	Checklist item	Page	Paragraph
**ADMINISTRATIVE INFORMATION**				
Title:				
Identification	1a	Identify the report as a protocol of a systematic review	1	1
Update	1b	If the protocol is for an update of a previous systematic review, identify as such	NA	NA
Registration	2	If registered, provide the name of the registry (such as PROSPERO) and registration number	NA	NA
Authors:				
Contact	3a	Provide name, institutional affiliation, e-mail address of all protocol authors; provide physical mailing address of corresponding author	1	1
Contributions	3b	Describe contributions of protocol authors and identify the guarantor of the review	2	3
Amendments	4	If the protocol represents an amendment of a previously completed or published protocol, identify as such and list changes; otherwise, state plan for documenting important protocol amendments	NA	NA
Support:				
Sources	5a	Indicate sources of financial or other support for the review	2	2
Sponsor	5b	Provide name for the review funder and/or sponsor	2	2
Role of sponsor or funder	5c	Describe roles of funder(s), sponsor(s), and/or institution(s), if any, in developing the protocol	2	2
**INTRODUCTION**				
Rationale	6	Describe the rationale for the review in the context of what is already known	5	2

For peer review only

Objectives	7	Provide an explicit statement of the question(s) the review will address with reference to participants, interventions, comparators, and outcomes (PICO)	5	3
**METHODS**				
Eligibility criteria	8	Specify the study characteristics (such as PICO, study design, setting, time frame) and report characteristics (such as years considered, language, publication status) to be used as criteria for eligibility for the review	6	6
Information sources	9	Describe all intended information sources (such as electronic databases, contact with study authors, trial registers or other grey literature sources) with planned dates of coverage	8	2
Search strategy	10	Present draft of search strategy to be used for at least one electronic database, including planned limits, such that it could be repeated	8	3
Study records:				
Data management	11a	Describe the mechanism(s) that will be used to manage records and data throughout the review	8	4
Selection process	11b	State the process that will be used for selecting studies (such as two independent reviewers) through each phase of the review (that is, screening, eligibility and inclusion in meta-analysis)	8	5
Data collection process	11c	Describe planned method of extracting data from reports (such as piloting forms, done independently, in duplicate), any processes for obtaining and confirming data from investigators	8	5
Data items	12	List and define all variables for which data will be sought (such as PICO items, funding sources), any pre-planned data assumptions and simplifications	NA	NA
Outcomes and prioritization	13	List and define all outcomes for which data will be sought, including prioritization of main and additional outcomes, with rationale	7	4
Risk of bias in individual studies	14	Describe anticipated methods for assessing risk of bias of individual studies, including whether this will be done at the outcome or study level, or both; state how this information will be used in data synthesis	9	2
Data synthesis	15a	Describe criteria under which study data will be quantitatively synthesised	9	3
	15b	If data are appropriate for quantitative synthesis, describe planned summary measures, methods of handling data and methods of combining data from studies, including any planned exploration of consistency (such as I^2, Kendall's τ)	NA	NA
	15c	Describe any proposed additional analyses (such as sensitivity or subgroup analyses, meta-regression)	NA	NA
	15d	If quantitative synthesis is not appropriate, describe the type of summary planned	NA	NA

For peer review only

Meta-bias(es)	16	Specify any planned assessment of meta-bias(es) (such as publication bias across studies, selective reporting within studies)	NA	NA
Confidence in cumulative evidence	17	Describe how the strength of the body of evidence will be assessed (such as GRADE)	NA	NA

From: Shamseer L, Moher D, Clarke M, Ghersi D, Liberati A, Petticrew M, Shekelle P, Stewart L, PRISMA-P Group. Preferred reporting items for systematic review and meta-analysis protocols (PRISMA-P) 2015: elaboration and explanation. BMJ. 2015 Jan 2;349(jan02 1):g7647

For peer review only
